# Optimizing Polyethylene Glycol Coating for Stealth Nanodiamonds

**DOI:** 10.1021/acsami.4c21303

**Published:** 2025-03-24

**Authors:** Edoardo Donadoni, Paulo Siani, Simone Gambari, Davide Campi, Giulia Frigerio, Cristiana Di Valentin

**Affiliations:** † Department of Materials Science, University of Milano-Bicocca, Via R. Cozzi 55, Milano 20125, Italy; ‡ BioNanoMedicine Center NANOMIB, University of Milano-Bicocca, Milano 20125, Italy

**Keywords:** nanodiamonds, PEGylation, titanium dioxide, nanoparticles, protein corona, nanomedicine, molecular dynamics

## Abstract

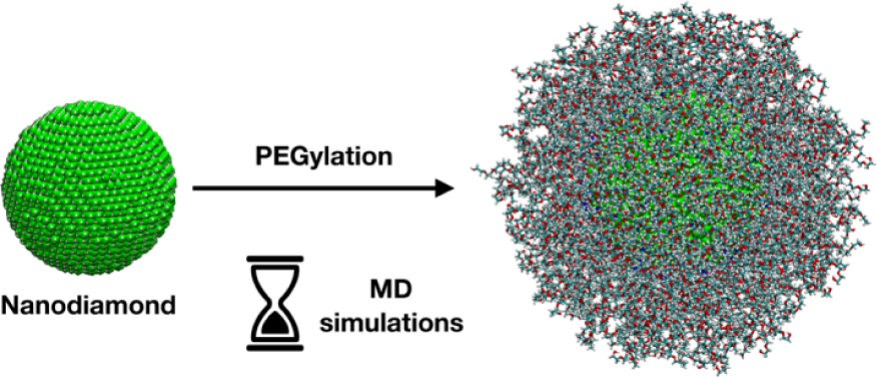

Nanodiamonds (NDs) have emerged as potential candidates for versatile platforms in nanomedicine, offering unique properties that enhance their utility in drug delivery, imaging, and therapeutic applications. To improve their biocompatibility and nanomedical applicability, NDs are coated with organic polymer chains, such as poly(ethylene glycol) (PEG), which are well known to prolong their blood-circulating lifetime by reducing the surface adsorption of serum proteins. Theoretical simulations are useful tools to define, at the atomic level, the optimal parameters that guide the presentation of the coating chains in the biological environment and the interaction of coated NDs with proteins. In this work, we perform atomistic molecular dynamics (MD) simulations of several PEGylated spherical ND models immersed in a realistic physiological medium. In particular, we evaluate the effect of the polymer chain’s terminal group, length, grafting density, and the ND core dimension on both the structural properties of the PEG coating and the interaction of the nanoconjugates with the aqueous phase. Moreover, we investigate the role played by the chemical nature of the core material through a comparative analysis with a PEGylated spherical titanium dioxide (TiO_2_) nanoparticle (NP). Among all the parameters evaluated, we find that the PEG grafting density, the PEG chain length, and the NP core material are key factors in determining the dynamic behavior of PEGylated nanosystems in solution, whereas the PEG terminal group and the ND dimension only play a marginal role. These factors can be strategically adjusted to identify the optimal conditions for enhanced clinical performance. Finally, we prove that the PEG coating prevents the aggregation of two ND particles. We believe that this computational study will provide valuable insights to the experimental community, supporting the rational design of polymer-coated inorganic NPs for more efficient nanomedical applications.

## 1 Introduction

Nanoparticles (NPs) have emerged in the last few decades as innovative tools in medicine, revolutionizing approaches to diagnosis, treatment, and prevention of diseases. Ranging from 1 to 100 nm in size, these materials possess unique physicochemical properties that enhance their interactions with biological systems.^1^ Their small size allows for improved cellular uptake and tissue penetration, making them particularly effective in targeting specific cells and delivering therapeutic agents directly to diseased sites.^2^

For NPs to be implemented in clinical practice, however, they must first remain in the circulatory system long enough to reach the target areas. In this context, the primary limiting factor is the adsorption of serum proteins (such as opsonins) onto the surface of the NPs, forming a protein corona. This is referred to as the opsonization process, which triggers an immune response resulting in the expulsion of the NPs from the body.^3^

An effective strategy to prevent protein corona formation is to make the NPs “stealth”, i.e., to protect their surface by coating them with organic polymer chains. In this regard, poly(ethylene glycol) (PEG) has proven to be an efficient agent for enhancing dispersibility and biocompatibility, thanks to its flexibility, hydrophilicity, and low toxicity.^4^

Among the many types of NPs,^5,6^ carbon-based inorganic NPs, such as carbon nanodots^7,8^ or graphene oxide nanosheets^9^ have garnered significant interest for nanomedical applications, including drug delivery, bioimaging, and active targeting of tumor cells. Nanodiamonds (NDs) have emerged as promising candidates due to their biocompatibility, high surface area, and tunable functionalization.^10,11^ Moreover, their small size, typically in the range of 1 to 100 nm, enables them to navigate biological environments effectively.^12^

One of the most significant advantages of NDs is their ease of functionalization with a variety of biomolecules, which improves their interaction with biological systems. For instance, surface modifications can be achieved through covalent bonding, allowing for the attachment of drugs, antibodies, or peptides. This functionalization not only improves drug solubility and stability but also enables the targeting of specific cells or tissues, thereby reducing off-target effects.^13^

Moreover, NDs have demonstrated excellent photostability and fluorescence properties, making them effective contrast agents for imaging techniques such as fluorescence microscopy and magnetic resonance imaging.^14^

Recent works have also highlighted the potential of NDs in cancer therapy, where they can be used to deliver chemotherapeutic agents directly to tumor sites, thus enhancing therapeutic efficacy while reducing systemic toxicity.^15^ Furthermore, their unique mechanical properties lend themselves to applications in regenerative medicine, where they can support cell growth and differentiation.^16^

Moreover, through *in vitro* analyses, Cigler et al. reported on the capability of polymer-coated shell-encapsulated NDs to penetrate human prostate cancer cell membranes, contrary to bare NDs,^17^ and to serve as colloidally and biologically stable small interfering RNA delivery systems with wide-ranging applications for RNA interference-based therapies.^18^

Finally, NDs coated with organic polymer chains, such as PEG or polyglycerol (PG), have been successfully synthesized^19−21^ and have resulted in reduced protein corona formation and macrophage cell uptake compared to bare NDs of *in vitro* experiments.^22^

Computational studies based on density functional theory (DFT) and density functional tight binding (DFTB) have explored the geometries and electronic properties of NDs.^23^ In addition, the stability of NDs has been assessed after oxygen^24^ or nitrogen^25^ surface functionalization.

Several classical molecular dynamics (MD) simulations have also been performed on bare or surface-functionalized NDs. For instance, Ge and Wang^26^ studied the association of ND models with lipid membranes, combining MD with umbrella sampling calculations and using a united-atom model. They also estimated the surface charge density of NDs from experimental zeta potentials using the Gouy–Chapman theory.^27^ Finally, Hughes and Walsh^28^ investigated the interaction of NDs with stearin triglyceride bilayers.

Furthermore, a few atomistic MD studies have focused on organic polymer-coated NDs for nanomedical applications. Specifically, in drug delivery, PEGylated spherical NDs have been exploited as carriers for the anticancer agents irinotecan and curcumin.^29^ Regarding PEG parametrization, we highlight a recent work by Ho et al.,^30^ in which they performed MD simulations on ethylene glycol oligomers using the GAFF force field. Finally, the colloidal stability of hyperbranched polyglycerol-grafted (100) surfaces has been examined.^31^

Optimizing the molecular features of the polymer coating is of the utmost importance to enhance the clinical applicability of NPs and theoretical simulations serve as powerful tools to accomplish this objective. In previous works by some of us, we simulated PEG-coated titanium dioxide (TiO_2_) NPs by means of quantum mechanical calculations based on DFT and DFTB^32,33^ and atomistic and coarse-grained classical MD, to study the behavior of these PEGylated NPs in a realistic physiological environment,^34,35^ including permeating cell membranes^36^ and their application in active targeting of tumors.^37−40^ The rationale for using TiO_2_ NPs lies in their exceptional photocatalytic properties, which allow them to convert UV–vis light energy into chemically active species for therapeutic applications, functioning as reactive oxygen species (ROS)-generating systems.^41^

In this work, we use atomistic MD simulations to investigate and compare the dynamical behavior of several PEGylated inorganic NPs (NDs or TiO_2_ NPs) in a realistic physiological medium, studying the impact of the coating polymer terminal group, grafting density, length, and NP core material type and dimension on the polymer structural properties and their interactions with the aqueous environment.

We envision that this computational study will be beneficial to the experimental community for the rational design of polymer-coated inorganic NPs with enhanced stealth properties for efficient nanomedical applications.

## 2 Computational Methods

### 2.1 Systems and Their Nomenclature

In this section, we introduce the various systems investigated and their nomenclature. They all consist of a spherical inorganic NP, either ND or TiO_2_, with different sizes (diameters of either ∼2 or ∼5 nm), covered with PEG polymer chains of different types. In particular, we considered chains of different molecular weights (PEG_500_, MW ∼ 500 Da; PEG_1000_, MW ∼ 1000 Da) and with different terminal groups (the end group binding the NP is either −CONH_2_ for NDs or −OH in the case of the TiO_2_ NP, and the other end group is either −OH or −CH_3_). The general nomenclature of the coated nanosystems is NP^a^-*n*PEG_b_-X, where *NP* is the core material = ND, TiO_2_; *a* is the NP dimension in nm = 2, 5; *n* is the number of PEG chains = 25, 50, 100, 360; *b* is the PEG molecular weight in Da = 500, 1000; and *X* is the solution terminal group = −OH, −CH_3_. The systems where 2 ND particles coated with 50 PEG_500_–OH are solvated in the same simulation box and initially far from each other or interacting are named 2ND^2^-50PEG_500_–OH^far^ and 2ND^2^-50PEG_500_–OH^close^, respectively. The generic nomenclature “PEG” and “NP” will be used whenever referring to or comparing multiple PEG chains or NP core systems.

### 2.2 Preparation of the Models

**Figure 1:**
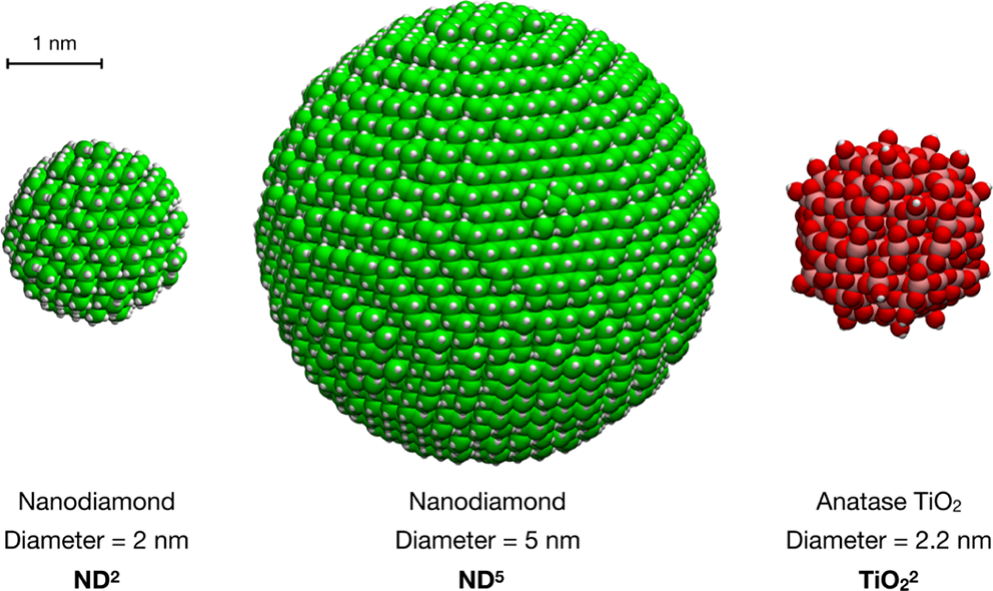
Structures of the bare ND and TiO_2_ NP systems. Carbon is shown in cyan, oxygen in red, nitrogen in blue, titanium in pink, and hydrogen in white. The carbon atoms of the ND core are shown in green.

We built the initial geometry of the bare ND models starting from the experimental lattice parameters and internal coordinates of bulk diamond, cutting ideal spheres with diameters of 2 nm and 5 nm (resulting in structures with 729 C atoms and 11543 C atoms, respectively, as shown in Figure 1).

For both models, we generated the final structure from molecular dynamics runs using a classical AIREBO^42^ potential to describe the interatomic interactions. We annealed and quenched each initial model at 500 K to reasonably mimic the thermal environment under experimental conditions. The models were then equilibrated for 1 ns at the target temperature, quenched down to 300 K in 100 ps, and equilibrated again at 300 K for 100 ps. A time step of 1 fs was used for the model generation process, and a Nosé-Hoover^43^ thermostat was employed during the equilibration process. Finally, we optimized the structure by performing a 0 K energy minimization with a 10^–10^ eV·Å^–1^ threshold on the forces. To test the reliability of our approach, for the smaller 729-C atom model we also performed the entire quenching, equilibration, and relaxation processes using a neural-network-based GAP^44^ potential, which provides a more accurate and general description of the carbon–carbon interactions. To temper the computational cost, we reduced the quenching and equilibration times by a factor of 10. This reduction does not significantly affect the final average geometry, as verified by comparing models obtained with AIREBO using both the longer and shorter generation processes. The final model produced using the GAP potential presents, on average, a morphology similar to that produced using AIREBO. In both cases, the LAMMPS^45^ code was used as a driver for molecular dynamics. The Quip^44^ module and TurboGAP^46^ were employed for the GAP potential.

We chose to use the models that were initially annealed at 500 K, as this temperature simulates the standard conditions used in experimental annealing. The next step was to graft the surface of the 2 or 5 nm ND models with either 25, 50, 100, or 360 PEG_500_ chains or PEG_1000_ chains with either −OH or −CH_3_ terminal groups, corresponding to a grafting density of 1.15, 2.3, or 4.6 chains·nm^–2^, by making covalent bonds between the N atoms of the −NH_2_ end group of PEG and the under-coordinated C atoms of the ND surface through an amide linkage, which is among the most commonly used experimentally to anchor PEG in a “grafting to” approach.^22^ Finally, we saturated the remaining under-coordinated surface C atoms of the NDs with H atoms.

The bare TiO_2_ NP model was designed by our group in previous works^32,33^ and consists of a spherical anatase TiO_2_ nanoparticle, carved from the crystalline bulk anatase structure and fully relaxed, first at the DFTB level of theory with a simulated annealing procedure, followed by a DFT optimization using the B3LYP hybrid functional. The stoichiometry of the NP is (TiO_2_)_223_·10H_2_O and it is characterized by an approximate diameter of 2.2 nm (Figure 1).

In the following studies,^34,35^ we grafted the surface of the TiO_2_ NP model with 50 methoxy-PEG_500_ polymer chains, whose −OH terminal group binds to 4-fold coordinated or 5-fold coordinated Ti atoms on the TiO_2_ NP surface, corresponding to a grafting density of 2.3 chains·nm^–2^.

### 2.3 Classical MD Simulations

The coated NDs were placed in cubic simulation boxes filled with mTIP3P^47^ water molecules using the GROMACS^48^ preparation tools (130 × 130 × 130 Å^3^ sized boxes for ND^2^-25PEG_500_–OH, ND^2^-50PEG_500_–OH, ND^2^-50PEG_500_-CH_3_, and ND^2^-100PEG_500_–OH; 150 × 150 × 150 Å^3^ sized boxes for 2ND^2^-50PEG_500_–OH^far^ and for 2ND^2^-50PEG_500_–OH^close^; and 180 × 180 × 180 Å^3^ sized boxes for ND^5^-360PEG_500_–OH and ND^5^-360PEG_1000_–OH). Na^+^ and Cl^–^ ions were added to neutralize the system charge and mimic the physiological concentration of 0.15 M. In Table S1, we report the exact composition of each simulated system model, including the number of water molecules and ions. All the systems were minimized using the steepest descent algorithm and then equilibrated for 1 ns at a constant temperature (303 K) and pressure (1 bar). The V-rescale thermostat^49^ with a coupling constant of 1.0 ps and the Parrinello–Rahman barostat^50^ with a coupling constant of 2.0 ps were used to control temperature and pressure. We employed the LINCS^51^ algorithm to constrain the bonds involving H atoms, and Newton’s equations of motion were integrated with the Velocity-Verlet leapfrog algorithm using a time step of 1.0 fs for a total production time of 100 ns. Long-range electrostatic interactions were handled with the particle mesh Ewald (PME)^52^ method with a cutoff distance of 12 Å, while short-range repulsive and attractive interactions were treated using the Lennard–Jones potential with a cutoff of 12 Å. Lennard–Jones combining rules were applied, and periodic boundary conditions (PBC) were imposed. The CGenFF^53^ parameters were employed to describe the bonded and nonbonded interactions in the PEGylated ND models. All minimization, equilibration, and production steps were performed using the open-source GPU-accelerated GROMACS^48^ code.

For the MD simulation of the TiO_2_-50PEG_500_-CH_3_ system, we used the LAMMPS^45^ package. The TiO_2_ NP was described by an improved Matsui-Akaogi FF, reparameterized by Brandt and Lyubartsev,^54^ while the CGenFF^47^ was employed for the adsorbed PEG chains. The FF used for the functionalized NP has been validated and employed in our previous works.^36−40,55−58^ The system topology was generated by means of the Moltemplate^59^ package for LAMMPS, and the system was immersed in a 100 × 100 × 100 Å^3^ mTIP3P^47^ water box, built with the PACKMOL^60^ software. During the simulation, we held the geometry of the NP core and the anchoring PEG −OH groups fixed at the DFTB-optimized geometry. We treated the NP as a thermalized rigid body, free to translate and rotate as a whole, with its internal degrees of freedom fixed at the DFTB-optimized geometry through the RIGID package in LAMMPS.^36−40,55^ This approach keeps the DFTB relative atomic positions within the TiO_2_ NP and avoids any mishap of the core during the MD simulation. In relation to the implications of this decision for the current study, which aims to examine the behavior of PEGylated NDs or TiO_2_ NPs in a physiological environment, treating the TiO_2_ NP core as a rigid entity has a minimal effect on both the PEG conformation and its interaction with water due to the dense PEG coating. The remaining degrees of freedom were free to evolve in time at 303 K (NVT ensemble), with a 2.0 fs time step, and the SHAKE algorithm imposed holonomic constraints on all the covalent bonds involving hydrogen atoms. PBC were used. Long-range electrostatic interactions were evaluated by the particle–particle particle-mesh (PPPM)^61^ solver, using a real-space cutoff of 12 Å. Short-range Lennard–Jones (12–6) interactions were smoothly truncated with a 12 Å cutoff by means of a switching function applied between 10 and 12 Å. Several energy minimization steps ensured that no atomic overlaps occurred, followed by an NVT equilibration and finally a production run of 100 ns.

### 2.4 Simulation Analysis

The last 10 ns of the 100 ns of each production simulation run were considered for analysis. VMD was used for graphical representations.^62^ The H-bonds were counted using the *gmx hbond* function of GROMACS according to the following geometrical criteria: (1) the distance between the H-bond donor and the H-bond acceptor heavy atoms is less than 3.0 Å; (2) the angle between the H-donor–acceptor is less than 20°. The nonbonded interaction energies were calculated using the *gmx energy* function of GROMACS. The mean radius of gyration (*R*_g_) of the polymer chains was calculated using the *gmx gyrate* function of GROMACS. In particular, *R*_g_ was computed as (eq 1)
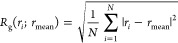
1where *r*_*i*_ and *r*_mean_ are the positions of the *i*-th atom and the center of mass of each PEG chain, and *N* is the total number of heavy atoms.

The mean end-to-end distance of the PEG chains, <*h*^2^>^1/2^, was computed as the average distance between the first and last heavy atoms of each PEG chain. The mean PEG–NP distance, *d*_PEG-NP_, was computed as the average distance between the last heavy atom of each PEG chain and the center of the NP.

The radial distribution function (RDF) and number density were obtained using the *gmx rdf* function of GROMACS, with a bin size of 0.1 Å and the appropriate normalization option. The polymer layer thickness, *thk*_PEG_, was computed from the normalized cumulative RDF of PEG as *r*_max_*– r*_min_, where *r*_min_ and *r*_max_ are radial distance values from the NP center such that RDF(*r*_min_) = 0.05 and RDF(*r*_max_) = 0.95, that is, the region where there is a 90% probability of finding the PEG layer, as previously described in other works.^63^

The polymer volume fraction is defined as the fraction of the total volume occupied by the polymer. It was calculated using 0.1 Å-wide spherical layers starting from the geometrical center of the NP. Each −CH_2_ group and O atom of PEG was assigned a volume of 20 Å^3^ and each water molecule a volume of 30 Å^3^, as done in previous works by some of us.^34,35,37^

The diffusion coefficient, *D*, of the PEGylated NDs and TiO_2_ NPs was estimated from the Einstein equation:
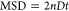
2where MSD is the mean-square displacement of atomic positions, *n* is the dimensionality of the diffusion, and *t* is the simulation time. In particular, the diffusion coefficients were obtained by fitting the MSD during the last 10 ns of the 100 ns-long MD simulations, where a linear dependency of the MSD with time was observed.

The total charge density around the NP (PEG coating + water + ions), σ(*r*), was computed by multiplying the number density of each atom type by its partial charge and summing them together. Then, the intrinsic electric field profile, *E*(*r*), was obtained from the total charge density through the Gauss law:
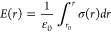
3where ε_0_ is the vacuum permittivity and the integration is performed from the NP surface, *r*_0_, to the bulk water phase.

Finally, the electrostatic potential, φ(*r*), was calculated by the integration of the electric field according to the following equation:
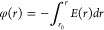
4

## 3 Results

The results are organized as follows: In Section 3.1 we analyze all the simulations in terms of the structural properties of the coating polymers; in Section 3.2, we focus on the interaction of the nanoconjugates with the surrounding aqueous environment; in Section 3.3, we estimate nanoconjugates’ self-diffusion coefficient and zeta potential; finally, in Section 3.4, we study the interaction of two coated ND systems. We refer to Section 4 for the discussion.

**Figure 2:**
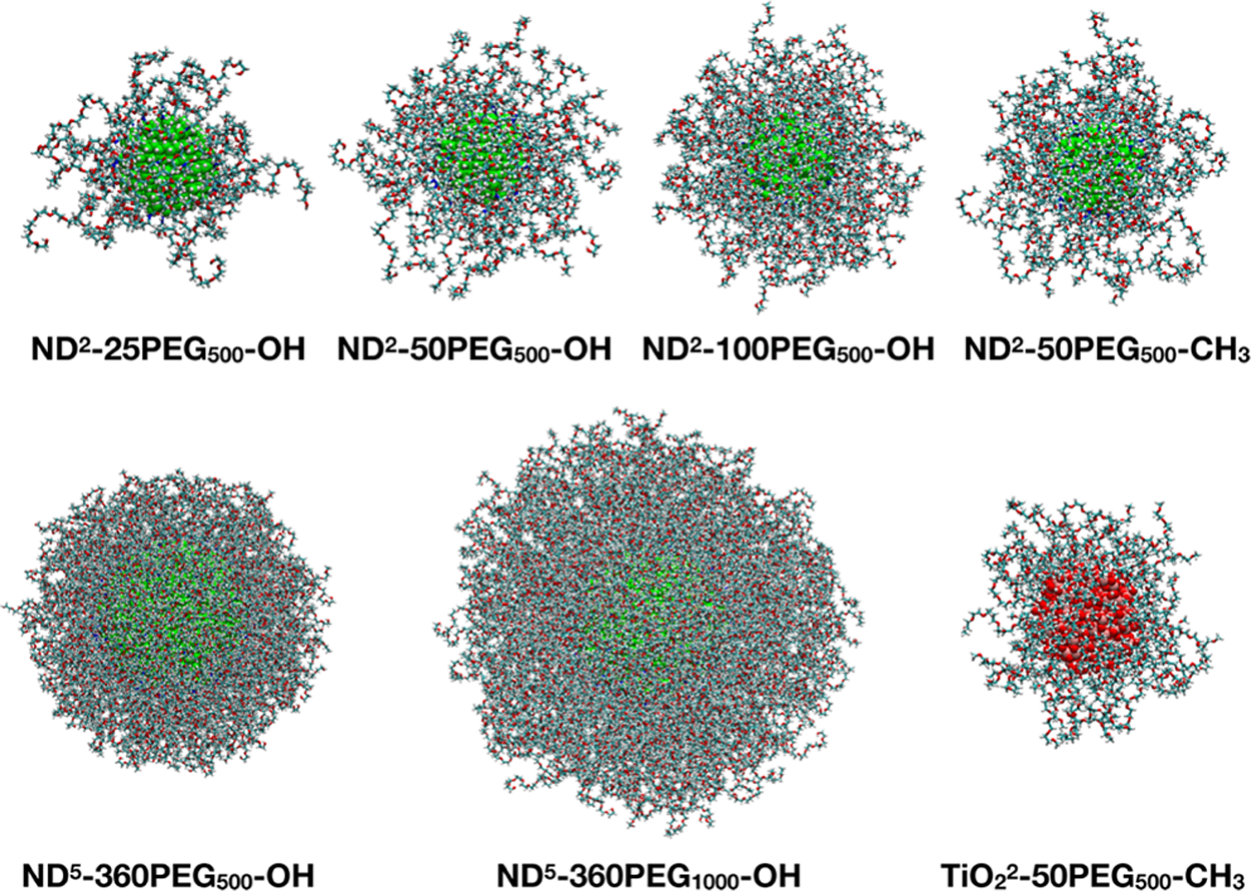
Last-frame snapshots from the 100 ns MD simulations of all of the investigated systems. Carbon is shown in cyan, oxygen in red, nitrogen in blue, titanium in pink, and hydrogen in white. The carbon atoms of the ND core are shown in green. The water molecules and the Na^+^ and Cl^–^ ions are not shown for clarity.

In Figure 2 the last-frame snapshots from the 100 ns MD simulations of all the investigated systems are shown.

### 3.1 Polymer Structural Properties

In this section, we report the results of the structural analysis related to the coating polymer chains, performed on the last 10 ns of the 100 ns MD simulations of all the investigated systems. In particular, in Table 1 we list the average radius of gyration (*R*_*g*_) of the polymer chains, end-to-end distance (<*h*^2^>^1/2^), PEG–NP distance (*d*_PEG-NP_) and thickness of the polymer layer (*thk*_*PEG*_). These quantities are introduced in Section 2.4.

**Table 1:** Average Radius of Gyration of the Polymer Chains, End-To-End Distance, PEG-NP Distance, and Thickness of the Polymer Layer Computed during the Last 10 ns of the 100 ns MD Simulations of Each System^a^

	ND^2^-25PEG_500_–OH	ND^2^-50PEG_500_–OH	ND^2^-100PEG_500_–OH	ND^2^-50PEG_500_-CH_3_	TiO_2_^2^-50PEG_500_-CH_3_	ND^5^-360PEG_500_–OH	ND^5^-360PEG_1000_–OH
*R*_g_ (Å)	6.4 (±0.1)	7.0 (±0.1)	7.6 (±0.1)	7.1 (±0.1)	6.7 (±0.1)	7.99 (±0.02)	12.40 (±0.04)
<*h*^2^>^1/2^ (Å)	16 (±2)	18 (±2)	21 (±2)	19 (±2)	17.0 (±0.5)	22 (±2)	36 (±3)
*d*_PEG-NP_ (Å)	22 (±3)	27 (±2)	31 (±2)	28 (±3)	27 (±2)	46 (±2)	60 (±3)
*thk*_PEG_ (Å)	12.46 (±0.03)	16.48 (±0.03)	20.18 (±0.03)	16.88 (±0.03)	15.17 (±0.03)	22.03 (±0.03)	34.84 (±0.03)

a In parentheses, the standard deviations are reported. A portion of the data relative to the TiO_2_^2^-50PEG_500_-CH_3_ system have been taken from a previous work by some of us.^37^

In general, we observe that <*h*^2^>^1/2^ and *d*_PEG-NP_ are associated with higher standard deviations with respect to *R*_g_ and *thk*_PEG_, which are more reliable quantities for estimating the extension and conformation of the polymer coating of nanometer-sized NPs. For further discussion on the effects of polymer structural properties influenced by different PEG chain terminal groups, lengths, grafting densities, and the material and dimensions of the NP core, we refer to Section 4.

**Figure 3:**
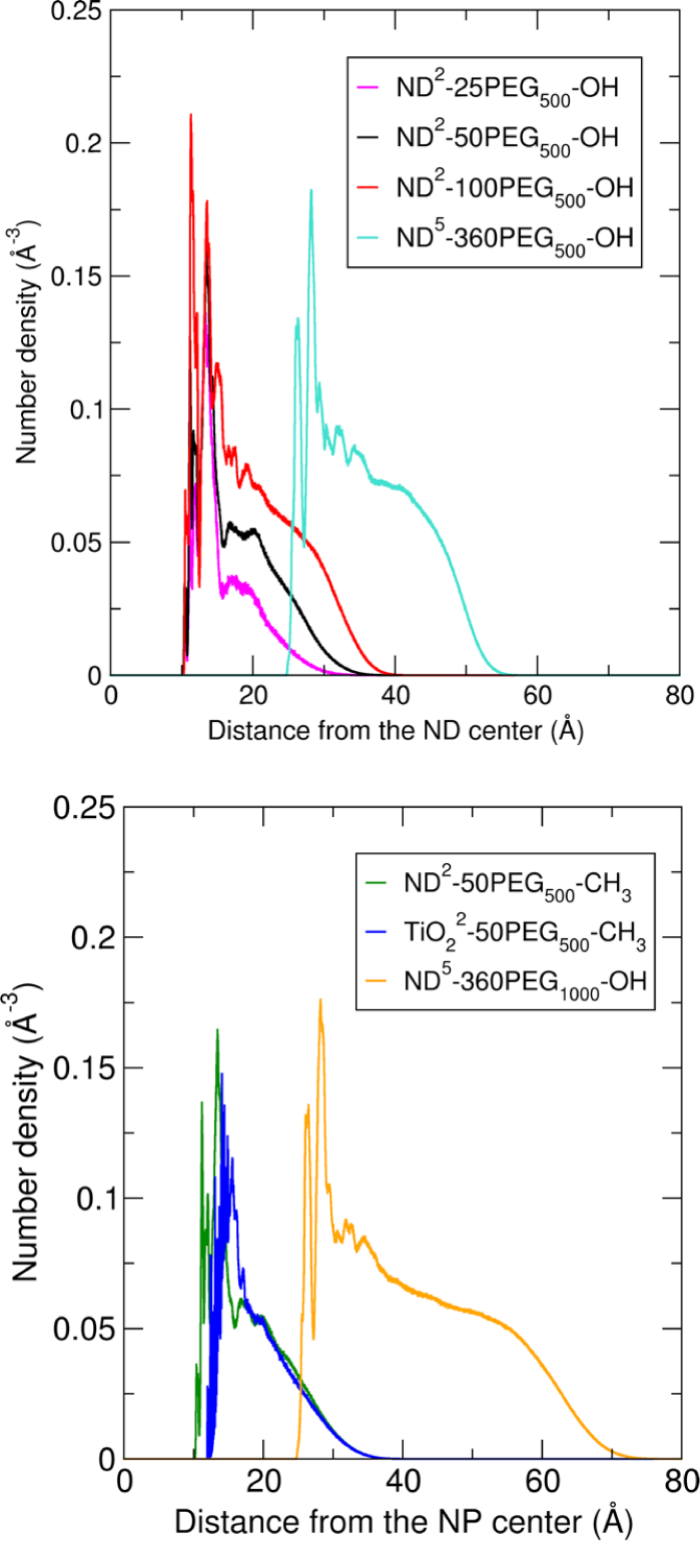
Average number density profiles of the PEG chains computed with respect to the central atom of the NP in the last 10 ns of the 100 ns MD simulations of every investigated system.

In Figure 3 we report the average number density profiles of the PEG chains computed with respect to the central atom of the NP.

At first sight, all the profiles have a similar shape, and they are set at different radial distances from the NP center depending on the NP core dimensions. Moreover, the intensity of the profiles depends on the length and density of the PEG chains. These effects are discussed in Sections 4.1–4.5.

In Table 2 the mean total NP/PEG chains and PEG/PEG chains nonbonded interaction energies, i.e., electrostatic + van der Waals (vdW), are listed for each system. Moreover, in Table S2, we report the average number of H-bonds among PEG chains (PEG/PEG).

**Table 2:** Average PEG/PEG and NP/PEG Nonbonded (Electrostatic + VdW) Interaction Energies Computed during the Last 10 ns of the 100 ns MD Simulations of Each System^a^

	ND^2^-25PEG_500_–OH	ND^2^-50PEG_500_–OH	ND^2^-100PEG_500_–OH	ND^2^-50PEG_500_-CH_3_	TiO_2_^2^-50PEG_500_-CH_3_	ND^5^-360PEG_500_–OH	ND^5^-360PEG_1000_–OH
Nonbonded interaction energy (kcal·mol^–1^)							
NP/PEG	–35 (±14)	218 (±23)	449(±23)	215 (±17)	–485 (±11)	3363 (±56)	3346 (±56)
PEG/PEG	–1459 (±27)	–3090 (±57)	–6042 (±95)	–1969 (±40)	–1262 (±23)	–24247 (±156)	–28589 (±156)

a In parentheses, the standard deviations are reported. A portion of the data relative to the TiO_2_^2^-50PEG_500_-CH_3_ system have been taken from a previous work by some of us.^37^

In general, we observe that the interaction of the PEG chains with the NP surface is energetically favorable only in the TiO_2_^2^-50PEG_500_-CH_3_ system for both electrostatic and van der Waals contributions, and slightly in the ND^2^-25PEG_500_–OH system for van der Waals forces only. In the case of the ND systems, the lack of NP/PEG chain interaction is compensated by a stronger interplay among PEG chains (PEG/PEG). For further discussion on the effect of the aforementioned parameters on the intermolecular interactions, we refer to Sections 4.1–4.5.

**Figure 4:**
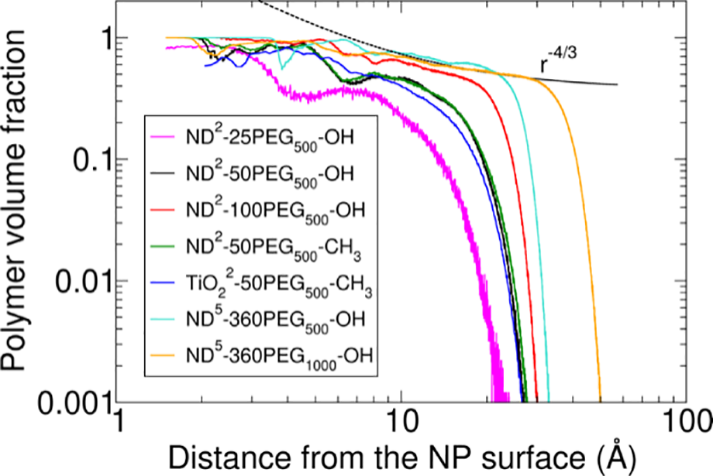
Log–log plot of MD predictions for the polymer volume fraction of PEG chains from the NP surface toward the bulk-water phase for all the investigated systems. The black dashed line corresponds to the Daoud-Cotton model prediction in the brush regime for the ND^5^-360PEG_1000_–OH system.

Finally, to examine the conformation of the polymer chains at the grafting density regimes used in this study (σ = 2.3 or 4.6 chains·nm^–2^), we compare our MD results with the theoretical predictions from the Daoud and Cotton model for polymer-coated NPs, as shown in Figure 4. This analytical model suggests that star-like polymers with a high grafting density have a central rigid core with a uniform polymer density. This regime is followed by a semidilute polymer brush one, where the polymer volume fraction behaves as *r*^*-4/3*^, with *r* being the distance from the NP surface (the fitting to the Daoud and Cotton model is shown in Figures S1–S7 with black dashed lines and also in Figure 4 for the ND^5^-360PEG_1000_–OH system).

The analysis of Figures 4 and S1–S7 shows that the brush regime predicted by the Daoud and Cotton model is confirmed by the MD predictions for the PEGylated NP models for systems coated with PEG_500_ and PEG_1000_ chains within the ranges of 8–18 Å and 15–28 Å from the NP surface, respectively. These findings confirm that low-weight PEG chains (500–1000 Da) attached to small, highly curved ND or TiO_2_ NPs at high grafting density can induce brush conformations extending a few nanometers from the NP surface. We note that previous work by some of us has already shown, through comparison with MD simulations, that the Daoud and Cotton model can correctly reproduce the behavior of coated systems with a grafting density of 2.3 chains·nm^–2^.^35^

### 3.2 Interaction with the Aqueous Environment

**Figure 5:**
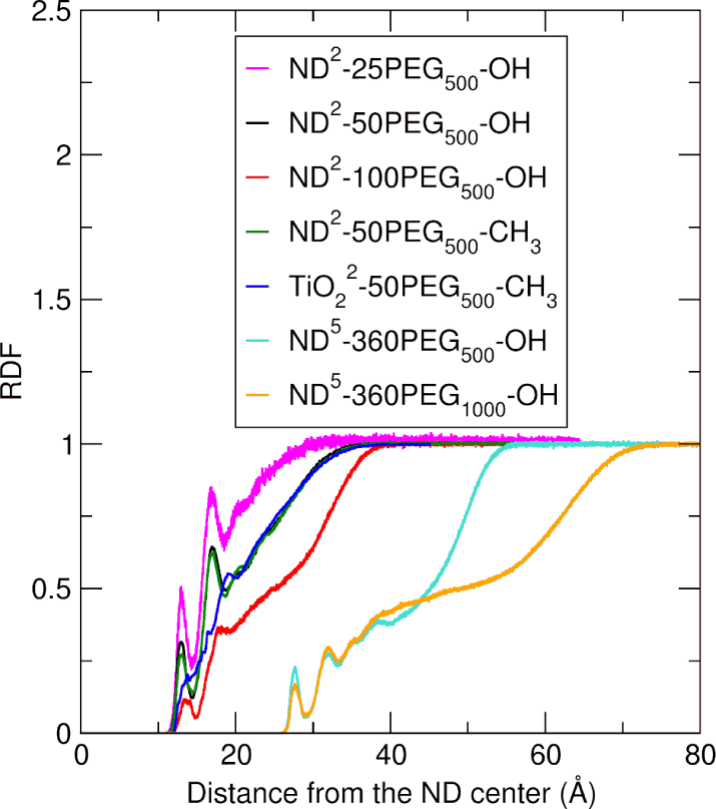
Average RDF of the water molecules computed with respect to the central atom of the NP on the last 10 ns of the 100 ns MD simulations of every investigated system.

The focus of this section is the study of the interaction of nanoconjugates with the physiological solution in which they are immersed. In Figure 5, the average radial distribution function of the water molecules with respect to the central atom of the NP is shown for every system. Moreover, by integrating the RDF profiles (Figure S8), we can estimate the number of water molecules at a given radial distance from the NP center.

At first glance, almost every profile is characterized by two relative maximum peaks in the region of the PEG chains, with an intensity lower than that of bulk water, demonstrating that the presence of the polymer coating limits the penetration of the water molecules in the region close to the NP surface. For further discussion on the spatial distribution of water molecules around the different PEGylated nanosystems, we refer to Sections 4.1–4.5.

Finally, in Table 3, we report the average nonbonded interaction energies (vdW + electrostatic) between the coated NPs and the aqueous medium. In Table S3, we also provide the average number of hydrogen bonds between the PEG chains and the water molecules (PEG/water).

**Table 3:** Average Nonbonded Interaction Energies between the Coated NPs and the Aqueous Phase during the Last 10 ns of the 100 ns MD Simulations of Each System^a^

	ND^2^-25PEG_500_–OH	ND^2^-50PEG_500_–OH	ND^2^-100PEG_500_–OH	ND^2^-50PEG_500_-CH_3_	TiO_2_^2^-50PEG_500_-CH_3_	ND^5^-360PEG_500_–OH	ND^5^-360PEG_1000_–OH
Nonbonded interaction energy (kcal·mol^–1^)							
NP/water	–37 (±9)	–3 (±5)	–5 (±1)	0 (±4)	–420 (±19)	–26 (±7)	5 (±7)
PEG/water	–3280 (±48)	–6109 (±117)	–10782 (±170)	–4609 (±177)	–5205 (±36)	–33022 (±240)	–74886 (±335)

a In parentheses, the standard deviations are reported. A portion of the data relative to the TiO_2_^2^-50PEG_500_-CH_3_ system have been taken from a previous work by some of us.^37^

In particular, from Table 3 we observe that the interaction energy between the NP and water is negligible in the case of the ND systems, whereas it is substantial for the TiO_2_ NP system. This outcome is reasonably correlated with the higher hydrophilic character of the TiO_2_ NP core compared to that of the hydrophobic NDs, which results in greater solvation in aqueous environments.

For further discussion on the interaction of the investigated nanosystems with the physiological medium, as a function of different coatings or NP core types/dimensions, we refer to Section 4.

### 3.3 Self-Diffusion and Zeta Potential

A key parameter for studying the dynamic behavior of the coated nanosystems is their self-diffusion coefficient. The estimated self-diffusion coefficients for all the systems are computed according to eq 2 and are presented in Table S4. Specifically, we observe that the D values range between 10^–10^ and 10^–11^ m^2^/s and are correlated with both the number and length of the PEG coating chains, as well as the mass and size of the nanoparticle core.

Finally, another interesting quantity that influences the water dispersibility of the coated nanosystems is their zeta potential, which is defined as the electrostatic potential at the shear plane between the relatively immobile and mobile layers of the solution adjacent to the solid surface. The zeta potential can be estimated from the electrostatic potential, which is computed according to eqs 3 and 4. The electrostatic potential, as a function of the radial distance from the NP center, is reported in Figure S9 for all systems under investigation.

Then, the zeta potential was estimated by setting the shear plane at a distance from the NP center where the bulk water phase begins, i.e., where the water RDF reaches 1 (Figure 5). In Table S5 we report the estimated zeta potential values for all the systems under study. In particular, we observe that the zeta potential is negative for the ND^2^-50PEG_500_-OH, ND^2^-50PEG_500_-CH_3_, and TiO_2_^2^-50PEG_500_-CH_3_ systems, while it is positive for the ND^5^-360PEG_500_-OH and ND^5^-360PEG_1000_–OH systems. The fact that some systems exhibit a positive zeta potential while others exhibit a negative zeta potential can be explained in terms of the total net charge density, which is the sum of Na^+^ and Cl^–^ ion charges at the shear plane (Figure S10). Specifically, the total net charge density at the shear plane is negative for ND^2^-50PEG_500_-OH, resulting in a negative zeta potential, while it is positive for ND^2^-100PEG_500_-OH, resulting in a positive zeta potential, which is likely an effect of a higher PEG grafting density on the NP.

For further discussion on the effects of the NP core and polymer coating features on zeta potential values, we refer to Section 4.

### 3.4 Interaction Between Two Coated ND Systems

**Figure 6:**
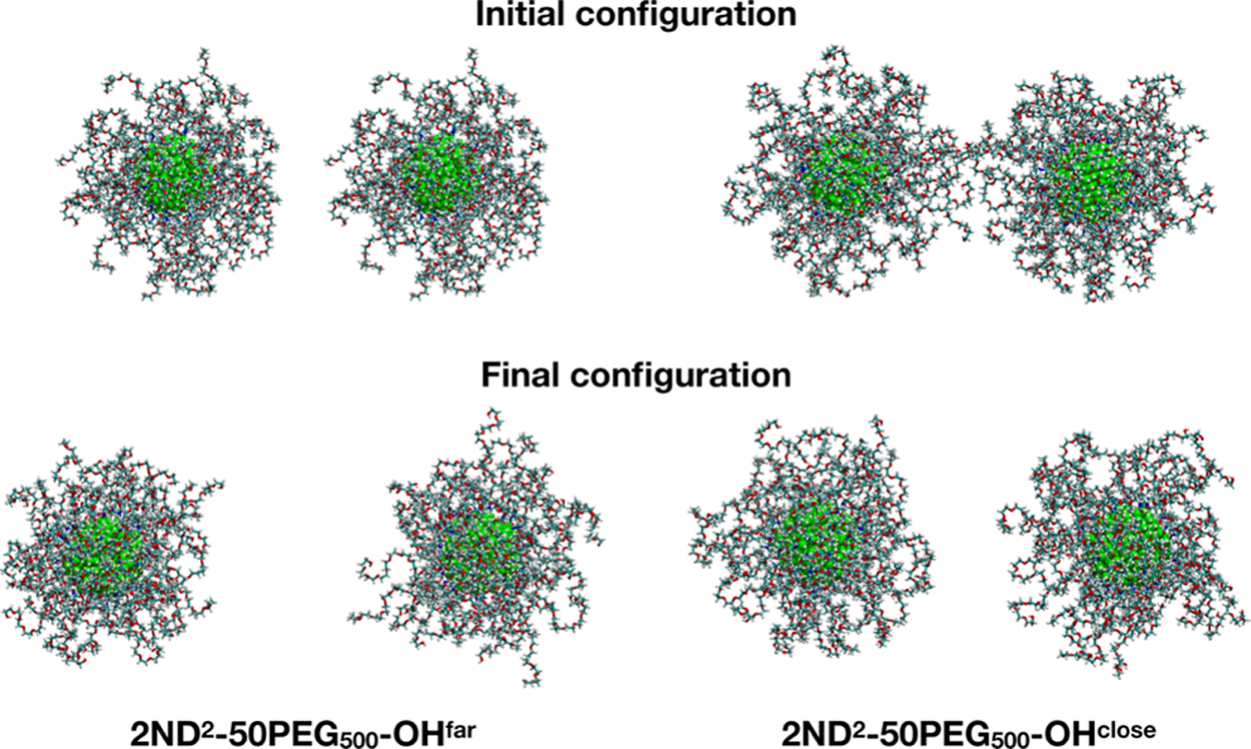
Initial and final snapshots from the 100 ns MD simulations of the 2ND^2^-50PEG_500_–OH^far^ and 2ND^2^-50PEG_500_–OH^close^ systems. Carbon is colored cyan, oxygen red, nitrogen blue, and hydrogen in white. The carbon atoms of the ND core are shown in green. The water molecules and the Na^+^ and Cl^–^ ions are not shown for clarity.

The role of the PEG coating is to enhance the biocompatibility of the nanoconjugates by minimizing their aggregation. To evaluate the impact of the polymer coating in preventing the nanosystems from interacting with each other, we performed two additional 100-ns-long MD simulations, where two ND particles, with a core dimension of 2 nm and coated with 50 PEG_500_-OH chains each, were immersed in the same 0.15 M NaCl water solution at 303 K and 1 bar. We chose to double the ND^2^-50PEG_500_–OH model because (i) the dimension of the ND cores (2 nm each) is feasible for an atomistic description of a double-sized system and (ii) the PEG grafting density is intermediate between the low (1.15 chains·nm^–2^) and the high grafting density (4.6 chains·nm^–2^) considered in the present study. Moreover, in order to remove a potential bias due to the starting-point configuration, we employed two different initial configurations: one where the coated NDs are separated by at least 1 nm from each other (2ND^2^-50PEG_500_–OH^far^) and the other where they are initially interacting (2ND^2^-50PEG_500_–OH^close^). From visual inspection of Figure 6, which shows the last-frame snapshots from the two MD simulations, and from the average intersystem interaction energy in Table S7, we demonstrate that the two coated ND systems do not aggregate, confirming the role of the PEG coating in reducing nanoparticle aggregation. We also performed an analogous structural and energetic analysis as in Section 3 for these new simulation data (Figures S11 and S12, Tables S6 and S7), which are in excellent agreement with the results for ND^2^-50PEG_500_–OH, further strengthening the robustness of our findings.

## 4 Discussion

In this section, we discuss the results shown previously through a comparative approach, evaluating the effect of the polymer terminal group (Section 4.1), the polymer grafting density (Section 4.2), the ND core dimension (Section 4.3), the polymer length (Section 4.4), and the NP core material (Section 4.5) on the structural and dynamic properties of coated NPs, as presented in Section 3 in light of recent experimental and theoretical findings as well.

### 4.1 Effect of the Polymer Terminal Group

In this section, we examine the effect of the polymer terminal group. In particular, we compare the ND^2^-50PEG_500_-**OH** and ND^2^-50PEG_500_-**CH**_**3**_ systems, which have the same polymer length, grafting density, and NP core dimension/type.

We notice from Table 1 that *R*_g_, <*h*^2^>^1/2^, *d*_PEG-NP_, and *thk*_PEG_ are not significantly affected by the change in the polymer terminal group, although we observe that the PEG–OH chains are closer to the ND surface and more coiled, resulting in a slightly lower thickness of the polymer layer (16.48 vs. 16.88 Å). Moreover, the PEG number density profiles in Figure 3 and the water RDF profiles in Figure 5 appear very similar for the ND^2^-50PEG_500_–OH (black) and the ND^2^-50PEG_500_-CH_3_ (green) systems, confirming the minimal effect of the terminal group on the polymer presentation.

Regarding the interaction energy of PEG with the solvent (Table 3), this is more negative in ND^2^-50PEG_500_–OH (−6109 vs −4609 kcal·mol^–1^) due to the more hydrophilic character of the –OH terminal group compared to the –CH_3_ one, making the ND^2^-50PEG_500_–OH system slightly more water-dispersible and therefore more suitable for nanomedical purposes. In this respect, we would also expect to observe a higher number of PEG/water H-bonds in Table S3 for ND^2^-50PEG_500_–OH than for ND^2^-50PEG_500_-CH_3_. However, the two mean values are comparable: 153 vs 151. Indeed, the −OH groups of the PEG–OH chains are responsible for 26 out of the total 153 H-bonds with water (i.e., 50% of the −OH groups interact with water via H-bonds, on average). This indicates that the other oxygen atoms of the PEG–OH chains form fewer H-bonds with water compared to the corresponding oxygen atoms in the PEG–CH_3_ chains. This is consistent with a more negative PEG/PEG interaction energy among the PEG–OH chains (−3090 kcal·mol^–1^) than among the PEG–CH_3_ ones (−1969 kcal·mol^–1^) (Table 2): the PEG–OH chains interact more strongly among themselves, reducing the thickness of the polymer layer (Table 1) at the expense of a reduced average number of H-bonds with the surrounding water.

Lastly, we do not observe any significant effect of the polymer terminal group on the self-diffusion coefficient or the estimated zeta potential value in Tables S4 and S5, respectively.

From the considerations above, we conclude that the chemical nature of the polymer chain terminal group does not impact considerably the presentation of the coating chains, except for some fine details. Moreover, changing the hydrophobicity/hydrophilicity of the terminal groups has little effect on the interaction with the aqueous phase. These outcomes align with the results of a recent work by some of us,^36^ where we found that the type of polymer terminal group of NP coating is not the most crucial parameter in determining the interaction of the nanoconjugates either with the physiological medium or with the lipid membrane into which they are incorporated.

### 4.2 Effect of the Polymer Grafting Density

Here, we analyze the effect of the polymer grafting density. For this purpose, we compare the ND^2^-**25**PEG_500_–OH, ND^2^-**50**PEG_500_–OH and ND^2^-**100**PEG_500_–OH systems, where the 2 nm ND is coated with either 25, 50, or 100 PEG_500_–OH chains (grafting densities of 1.15, 2.3, and 4.6 chains·nm^–2^, respectively).

By doubling the number of grafted PEG chains, we observe that the magnitude of the PEG/PEG interaction energy doubles (−1459 vs. −3090 vs. −6042 kcal·mol^–1^ in Table 2), as expected. Moreover, because of the increased concentration of attached PEG chains on the ND, there is less space for them to coil, as demonstrated by greater *R*_g_, <*h*^2^>^1/2^, *d*_PEG-NP_, and *thk*_PEG_ values for ND^2^-100PEG_500_–OH than for ND^2^-50PEG_500_–OH (9%, 17%, 15%, and 22%, respectively) and for ND^2^-25PEG_500_–OH (19%, 31%, 41%, and 62%, respectively) (Table 1). The increase in the thickness of the polymer layer is also evident from the PEG number density profiles in Figure 3, as well as from the polymer volume fraction profiles in Figure 4, where, in both cases, the profile of the ND^2^-100PEG_500_–OH system (in red) decays to zero at higher distances from the ND center compared to the ND^2^-50PEG_500_–OH system (in black) and the ND^2^-25PEG_500_–OH system (in magenta).

Regarding the interaction with the aqueous environment, we observe a decrease in the intensity of the water RDF profile of ND^2^-100PEG_500_–OH at the position of the first two solvation shells and, in general, in the PEG region (Figure 5). This is a result of a denser polymer coating around the ND compared to the ND^2^-50PEG_500_–OH and ND^2^-25PEG_500_–OH systems. In particular, the reduced content of water molecules inside the polymer coating layer in the ND^2^-100PEG_500_–OH system results in the PEG/water interaction energy and the number of H-bonds being less than the expected double of those for ND^2^-50PEG_500_–OH (Table 3, −10782 kcal·mol^–1^ vs. −6109 kcal·mol^–1^ for the interaction energy; Table S3, 262 vs. 153 for the number of H-bonds) and less than the expected four times of those for ND^2^-25PEG_500_–OH (Table 3, −10782 kcal·mol^–1^ vs. −3280 kcal·mol^–1^ for the interaction energy; Table S3, 262 vs. 78 for the number of H-bonds).

The self-diffusion coefficient values in Table S4 inversely correlate with the polymer grafting density, especially when transitioning from ND^2^-50PEG_500_–OH to ND^2^-100PEG_500_–OH system, as the mass of the nanosystem increases with higher grafting densities. Conversely, the estimated zeta potential values in Table S5 do not exhibit any significant correlation with the number of PEG chains grafted onto the nanoparticle surface.

Our results have shown that a change in the polymer grafting density has a remarkable influence on the polymer’s structural properties and on the interaction of the nanoconjugates with the physiological medium. In particular, doubling the grafting density results in an increase in the average values of the polymer chain’s *R*_g_, <*h*^2^>^1/2^, and *d*_PEG-NP_, as well as in *thk*_PEG_, the polymer layer thickness, in agreement with the results of previous works by some of us on PEGylated TiO_2_ NP systems.^34,35^ Moreover, the higher concentration of chains on the ND surface reduces the number of water molecules that can penetrate the coating.

The increase in the polymer layer thickness with the increase in the polymer grafting density was also observed experimentally by Nishikawa et al.^21,22^ on 5–50 nm-sized spherical NDs coated with polyglycerol (PG). Additionally, Zou et al.^22^ found that both protein corona adsorption and macrophage uptake decrease with the increase in PG content for 100 nm-sized PG-grafted NDs or superparamagnetic iron oxide nanoparticles (SPIONs), suggesting that a thicker polymer layer could more efficiently shield the NPs from serum proteins and, therefore, enhance their biocompatibility and nanomedical applicability.

### 4.3 Effect of the NP Core Dimension

In this section, we study the effect of the NP core dimension. With this aim, we now compare the **ND**^**2**^-100PEG_500_–OH and **ND**^**5**^-360PEG_500_–OH systems, for which, respectively, a 2 nm or 5 nm ND is coated with either 100 or 360 PEG_500_ chains. Hence, these two systems are coated with PEG chains of the same length and with the same terminal group, both resulting in a grafting density of 4.6 chains·nm^–2^.

Results in Table 1 indicate that increasing the dimension of the ND core, and thus reducing the surface curvature, results in a moderate increase in the PEG radius of gyration (5%), end-to-end distance (5%) and layer thickness (9%) values. On the contrary, Table 3 shows a 15% decrease in the PEG/water nonbonded interaction energy, after normalization against the number of PEG chains, when going from ND^2^-100PEG_500_–OH to ND^5^-360PEG_500_–OH. This decrease correlates with the increase in the PEG/PEG chain interaction for the latter system (Table 2).

Moreover, an increase in the mass of the ND slows down the diffusion of the nanosystem (Table S4) but does not affect its zeta potential (Table S5).

In light of the previous considerations, we conclude that the dimension of the NP core, provided the same PEG length and grafting density, does not significantly affect the polymer presentation and, therefore, we can assume that it is not a crucial parameter that would influence protein corona formation.

For instance, Zou et al.^22^ experimentally confirmed that human plasma corona protein adsorption, together with U937 macrophage cell uptake, is not impacted by the dimension of the NP (30, 50, or 100 nm-sized NDs or SPIONs), but depends on the polymer grafting density, as discussed in the previous section, and on the polymer type (PEG or PG).

NPs with core dimensions between 1 and 100 nm are exploited in nanomedicine because they are small enough to be either excreted by renal filtration (diameter <5.5 nm) or accumulated within organs associated with the mononuclear phagocyte system, primarily the spleen and liver.^64^ Although larger NPs (up to 100 nm) have a more realistic size from the experimental point of view compared to those we have simulated (2–5 nm), such extensive systems, especially when considering the polymer coating, can only be studied using more affordable computational methods, such as coarse-graining techniques, which come with the trade-off of decreased chemical accuracy and a loss of detailed atomistic representation.

### 4.4 Effect of the Polymer Length

In this paragraph, we discuss the effect of the length of the PEG chains by comparing the ND^5^-360**PEG**_**500**_–OH and ND^5^-360**PEG**_**1000**_–OH systems, where larger NDs of 5 nm size are coated with 360 PEG_500_–OH or PEG_1000_–OH chains, i.e., with the same terminal group and grafting density (4.6 chains·nm^–2^).

Clearly, the increase in the polymer chain length makes the PEG number density profile extend toward higher distances from the NP center (Figure 3, orange vs. light blue plot). In particular, when transitioning from the ND^5^-360PEG_500_–OH to the ND^5^-360PEG_1000_–OH system, the doubling of the PEG length results in increases of 55%, 64%, 30%, and 58% in the values of *R*_g_, <*h*^2^>^1/2^, *d*_PEG-NP_, and *thk*_PEG_, respectively. This indicates that the values of the polymer structural quantities do not increase linearly with the increase in the PEG chain length, proving that PEG_1000_–OH chains are relatively more coiled than PEG_500_–OH chains with shorter lengths. Also, the average *R*_g_ value for PEG_1000_–OH chains is in fair agreement with those computed in a previous work by some of us^36^ for PEG_1000_ chains with either methyl, deprotonated carboxyl, or protonated amine terminal groups attached to a spherical TiO_2_ NP, at both atomistic and coarse-grained levels.

Regarding the nature of the molecular interactions, we observe a moderate increase in the magnitude of the PEG/PEG chain interaction energy (18%, Table 2) when transitioning from the ND^5^-360PEG_500_–OH to the ND^5^-360PEG_1000_–OH system, while, on the contrary, the PEG/water interactions increase by 127% (Table 3).

Finally, in Figure 5, the RDF profiles of water for ND^5^-360PEG_500_–OH (orange) and ND^5^-360PEG_1000_–OH (light blue) have a very similar shape. Moreover, the intensity of the RDF peak corresponding to the first solvation shell (at a radial distance of about 28 Å from the ND center) is slightly less intense for ND^5^-360PEG_1000_–OH than for ND^5^-360PEG_500_–OH, indicating a lower density of water molecules in the region near the ND surface, while the opposite trend is observed for the second solvation shell.

Clearly, doubling the length of the PEG chains results in a decreased self-diffusion coefficient, as shown in Table S4, due to the effect of the mass increase, which is in line with the discussion above. The zeta potential values in Table S5 do not exhibit a significant trend.

Based on the points mentioned, coating NDs with PEG_1000_–OH chains instead of PEG_500_–OH chains, i.e., increasing the PEG length by 100%, evidently results in an increase in polymer thickness, which, however, is less than 100%. This indicates that PEG_1000_–OH chains are proportionally more coiled than the PEG_500_–OH chains. Moreover, PEG_1000_–OH chains provide higher crowdedness on the ND surface and also an increase in the polymer layer thickness compared to PEG_500_–OH chains, suggesting that protein adsorption on ND may be reduced, as experimentally observed.^22^ Additionally, the higher solvation of the PEG_1000_–OH chains compared to the PEG_500_–OH ones may limit protein corona formation,^65^ as PEG/water interactions are likely more favorable than PEG/protein ones. Indeed, it has been proposed that the interaction between PEG and water forms a barrier of solvent between the NP surface and proteins, hindering their interaction.^66^

Numerous experimental studies have shown that moderate PEGylation (with PEG chains of 2000 Da in molecular weight) provides the best balance between preventing opsonization and promoting cellular uptake,^4^ although this remains a topic of debate in the experimental literature.^67,68^ However, atomistic MD simulations of PEGylated NDs with longer PEG chains, especially in the presence of serum proteins, would become challenging for current state-of-the-art computational resources to achieve satisfactory sampling of the phase space. Computational approaches with low resolution, such as coarse-graining methods, are most suitable for handling such large systems, as demonstrated in a previous work by some of us to study the interaction of polymer-coated inorganic NPs with lipid membranes.^36^ Nevertheless, some experimental studies have also found that shorter PEG chains with low molecular weight (i.e., PEG_350_) exhibit similar circulation lifetimes as the longer and heavier ones do (i.e., PEG_2000_)^67^ and may enhance cellular uptake in breast cancer and myeloma cells^68^ making our model study more relevant.

### 4.5 Effect of the NP Core Material Type

Finally, to understand the role played by the NP core material type on the polymer coating structure and its interaction with the physiological medium, in this section, we compare the results of structural and dynamic quantities computed from MD simulations of **ND**^**2**^-50PEG_500_-CH_3_ and **TiO**_**2**_^**2**^-50PEG_500_-CH_3_ systems, where a ND or TiO_2_ spherical NP with an approximate diameter of 2 nm is grafted with 50 PEG_500_-CH_3_ chains (grafting density of 2.3 chains·nm^–2^). The purpose of the comparison is to examine how the different polarity of the core (hydrophobic carbonaceous ND vs. hydrophilic TiO_2_ NP) affects the study of the conformational behavior of attached coating chains and the distribution of surrounding water molecules.

At first, we observe from Figure 3 that the shapes of the PEG number density profiles (green for ND^2^-50PEG_500_-CH_3_ and blue for TiO_2_^2^-50PEG_500_-CH_3_ systems) are similar. In particular, the profile for TiO_2_^2^-50PEG_500_-CH_3_ is shifted toward higher distances (1.7 Å) from the NP center compared to that of ND^2^-50PEG_500_-CH_3_, and this is due to the different anchoring bond types (amide bond for ND^2^-50PEG_500_-CH_3_ and undissociated coordinative bond for TiO_2_^2^-50PEG_500_-CH_3_) and also to the slightly greater size of the TiO_2_ NP with respect to the ND. Moreover, for TiO_2_-50PEG_500_-CH_3_ the PEG profile decays to zero more rapidly than that of ND^2^-50PEG_500_-CH_3_. This result is in agreement with the reduced values of *R*_g_, <*h*^2^>^1/2^, *d*_PEG-NP_, and *thk*_PEG_ found for TiO_2_^2^-50PEG_500_-CH_3_ compared to ND^2^-50PEG_500_-CH_3_ (6%, 11%, 4%, and 10%, respectively, Table 1). This behavior is rationalized by a more favorable interaction of the PEG chains with the NP surface and, conversely, reduced PEG/PEG interactions in the case of TiO_2_^2^-50PEG_500_-CH_3_, as shown in Table 2. This is a consequence of the more hydrophilic character of the TiO_2_ NP, whose surface titanium atoms can favorably interact with PEG oxygen atoms, compared with the ND surface considered in this work, where all surface carboxyl groups have been turned into amide groups to anchor PEG chains. It is reasonable to suppose that in real experiments, the extent of oxidation could be larger than the actual resulting PEG grafting density. In this case, the residual carboxyl groups, if protonated, would enhance the interaction with the PEG chains only if they were protonated as –COOH, so that they could establish H-bonds with the PEG O atoms.

Regarding the interaction of the nanoconjugates with the physiological solution, we observe, from Table 3, a weaker interaction of the PEG chains with water in the case of TiO_2_^2^-50PEG_500_-CH_3_ compared to ND^2^-50PEG_500_-CH_3_. This correlates with the stronger NP/PEG chain interaction observed for the TiO_2_^2^-50PEG_500_-CH_3_ system, as discussed earlier. The enhanced interaction of the PEG chains with the TiO_2_ NP surface makes it spatially less accessible to water molecules compared to the ND surface, as demonstrated by the intensity of the water RDF peaks in Figure 5 and the cumulative number of water molecules in Figure S8 (blue vs. green curves) within the region of the PEG coating (up to about 20 Å from the ND or TiO_2_ NP center). However, the metal oxide surface is electrostatically more attractive to water molecules compared to the ND surface, which results in a more negative NP/water interaction energy for TiO_2_^2^-50PEG_500_-CH_3_ than for ND^2^-50PEG_500_-CH_3_, as shown in Table 3. Furthermore, comparing in Figure 5 the water RDF profiles of ND^2^-50PEG_500_-CH_3_ (green) and TiO_2_-50PEG_500_-CH_3_ (blue), we observe that while the former is characterized by two distinct relative maximum peaks (at approximately 13 and 17 Å from the NP center), the latter does not exhibit any defined peaks but instead shows a more uniform trend. This difference suggests a more structured distribution of water molecules around the ND compared to the TiO_2_ NP, as illustrated in Figure S13, where the water molecules comprising the first and second solvation shells of the ND^2^-50PEG_500_-CH_3_ and TiO_2_^2^-50PEG_500_-CH_3_ systems are depicted. While it is true that more hydrophilic NPs, such as TiO_2_-based ones, should promote a more structured arrangement of water molecules (due to the potential for hydrogen bonding between water and the NP surface), it is also reasonable to conclude that the PEG chains—particularly the anchoring amide groups in ND^2^-50PEG_500_-CH_3_—contribute to the ordering of solvent molecules.

From the self-diffusion coefficients in Table S4 we notice that the heavier and more hydrophilic TiO_2_ nanosystem diffuses more slowly than the ND one, even though they expose the same polymer coating and share a similar zeta potential value (Table S5).

Given the discussion above, we can state that the chemical nature of the NP core material influences the extent of interaction between the NP and the PEG chains. In particular, a more hydrophilic NP, such as a TiO_2_-based one, has a stronger interaction with PEG, which results in the shrinking of the coating polymer layer. Moreover, different NP core materials determine a different distribution of the water molecules around the NP surface. The characteristics of water layers around the NP may influence the degree of protein corona formation, as the protein amino acids must replace the water molecules of the NP solvation shell to adsorb on the NP surface,^69^ although this still remains a challenging topic in nanotechnology.

## 5 Conclusions

PEGylation is a widely recognized approach to enhance the biocompatibility of nanomedical devices and has shown effectiveness in the case of numerous inorganic NPs, including NDs, which have recently emerged as promising platforms for diverse nanomedical procedures.

In this work, by means of atomistic MD simulations, we have unveiled the impact of several parameters, namely the terminal group, length, and grafting density of the PEG chains, as well as the material type and dimension of the NP core, on the dynamics of PEG-grafted NDs or TiO_2_ NPs in a realistic physiological environment through a comparative analysis.

These parameters have been shown to affect the presentation of the PEG chains and their interaction with the NP, other PEG chains, and the aqueous phase to varying degrees and extents. In particular, different PEG terminal groups do not influence the polymer radius of gyration or thickness but only the PEG/PEG and PEG/water interactions, making nanosystems coated with PEG–OH chains slightly more water-soluble and, hence, more suitable for clinical applications. Conversely, increasing the PEG chain length or grafting density results in a thicker and more solvated polymer layer, which could reduce protein corona formation on the NP surface. Finally, we have observed that the chemical nature of the NP core dictates the strength of the NP/PEG interactions and the distribution of the solvent around the NP. Specifically, the TiO_2_ NP interacts more favorably with PEG due to its more pronounced hydrophilic character compared to the ND.

Therefore, we can deduce that, among all the parameters considered, the PEG grafting density, PEG length, and the NP core material have the greatest impact on the behavior of PEGylated nanosystems in solution and thus can be strategically tuned to find the optimal settings for more performant nanomedical devices, especially with regard to tailoring the protein corona. Finally, we proved that PEG coating prevents the aggregation of two ND particles.

In conclusion, we believe that our work not only advances the computational simulation of polymer-coated inorganic NPs, but also offers valuable guidelines to the experimental community for the rational design of PEGylated nanosystems with enhanced biocompatibility for effective clinical applications.

## Supporting Information Available

System’s composition; number of PEG/PEG and PEG/water hydrogen bonds; log–log plots of polymer volume fraction; count of water molecules in the PEG coating; self-diffusion coefficients of nanoconjugates; electrostatic potential and estimated zeta potential of nanoconjugates; data analysis of 2ND^2^-50PEG_500_–OH^far^ and 2ND^2^-50PEG_500_–OH^close^ systems; and first and second solvation shells of the ND^2^-50PEG_500_-CH_3_ and the TiO_2_^2^-50PEG_500_-CH_3_ systems (PDF)
