# Supplementary material for: Optimizing Polyethylene Glycol Coating for Stealth Nanodiamonds
Source: ACS Appl Mater Interfaces. 2025 Mar 24;17(13):19304–16. doi: 10.1021/acsami.4c21303 (PMC11969435; doi:10.1021/acsami.4c21303)
Supplement: Supplementary file 1 — am4c21303_si_001.pdf [file am4c21303_si_001.pdf]

Supporting Information

Optimizing polyethylene glycol coating for stealth nanodiamonds

Edoardo Donadoni,[a,b] Paulo Siani,[a,b] Simone Gambari,[a] Davide Campi,[a] Giulia Frigerio,[a,b] Cristiana Di Valentin[a,b*]

[a] *Department of Materials Science, University of Milano-Bicocca, Via R. Cozzi 55, I-20125, Milano, Italy*

[b] *BioNanoMedicine Center NANOMIB, University of Milano-Bicocca, Italy*

[*]*Corresponding author: cristiana.divalentin@unimib.it*

Table S1. Composition of each simulated system in terms of water molecules and ions.

System	Number of water molecules	Number of ions
ND^2-25PEG_{500}-OH	70359	396
ND^2-50PEG_{500}-OH	70561	396
ND^2-100PEG_{500}-OH	68994	396
ND^2-50PEG_{500}-CH_3	70526	396
$TiO_2{}^2$-50PEG_{500}-CH_3	64817	0
ND^5-360PEG_{500}-OH	176881	1054
ND^5-360PEG_{1000}-OH	167492	1054
2ND^2-50PEG_{500}-OH^{far}	104644	610
2ND^2-50PEG_{500}-OH^{close}	104657	610

Table S2. Average PEG/PEG H-bonds number computed on the last 10 ns of the 100 ns MD simulations of each system. In parenthesis, the standard deviations are reported.

	ND^2-25PEG_{500}-OH	ND^2-50PEG_{500}-OH	ND^2-100PEG_{500}-OH	ND^2-50PEG_{500}-CH_3	$TiO_2{}^2$-50PEG_{500}-CH_3	ND^5-360PEG_{500}-OH	ND^5-360PEG_{1000}-OH
H-bonds number							
PEG/PEG	0.5 (±0.1)	1.3 (±1.6)	0.6 (±1.1)	1.0 (±1.4)	2 (±1)	33 (±6)	32 (±6)

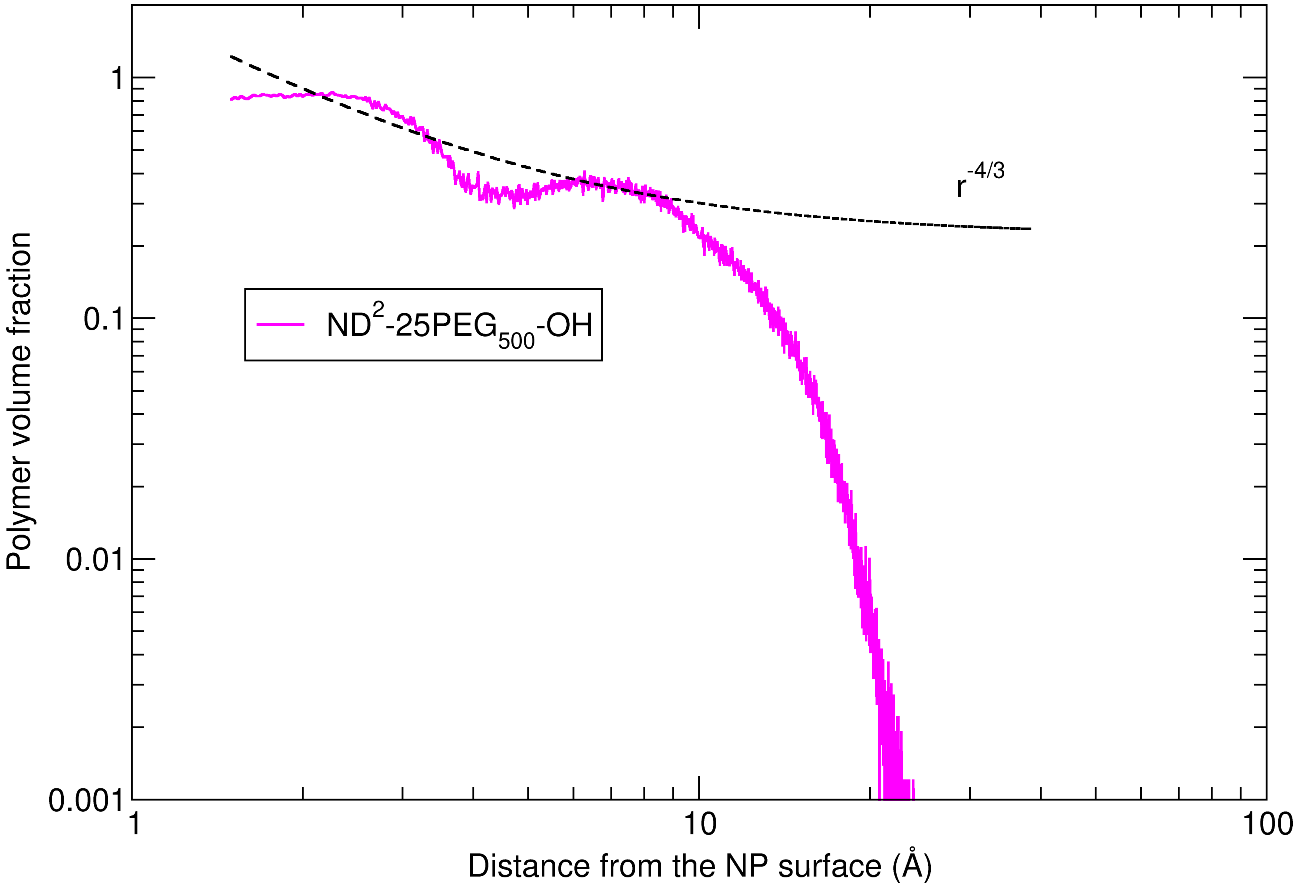

Figure S1. Log-log plot of MD predictions for the polymer volume fraction of the PEG chains from the NP surface towards the bulk-water phase for the ND^2-25PEG_{500}-OH system. The black dashed line corresponds to the Daoud-Cotton model prediction in the brush regime.

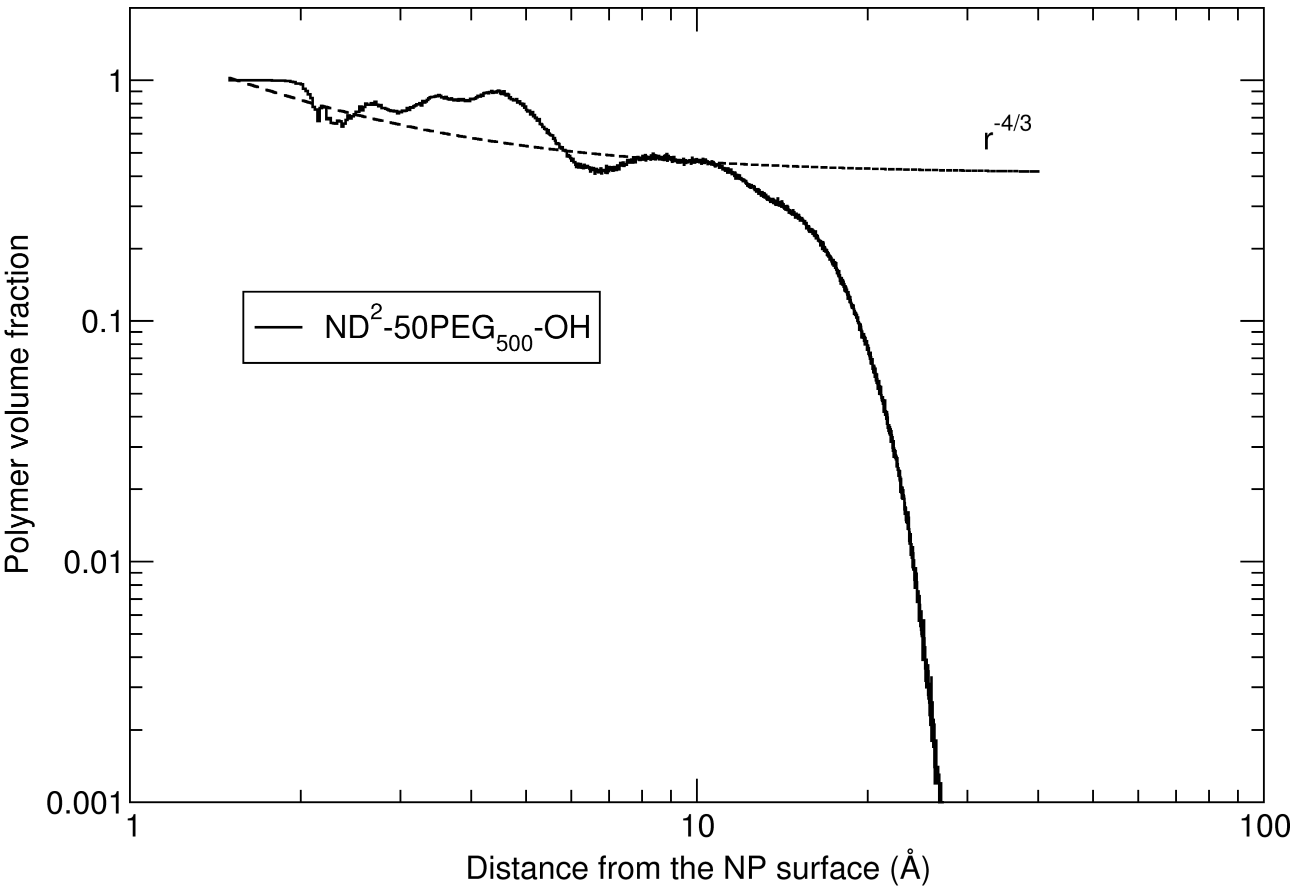

Figure S2. Log-log plot of MD predictions for the polymer volume fraction of the PEG chains from the NP surface towards the bulk-water phase for the ND^2-$50PEG_{500}$-OH system. The black dashed line corresponds to the Daoud-Cotton model prediction in the brush regime.

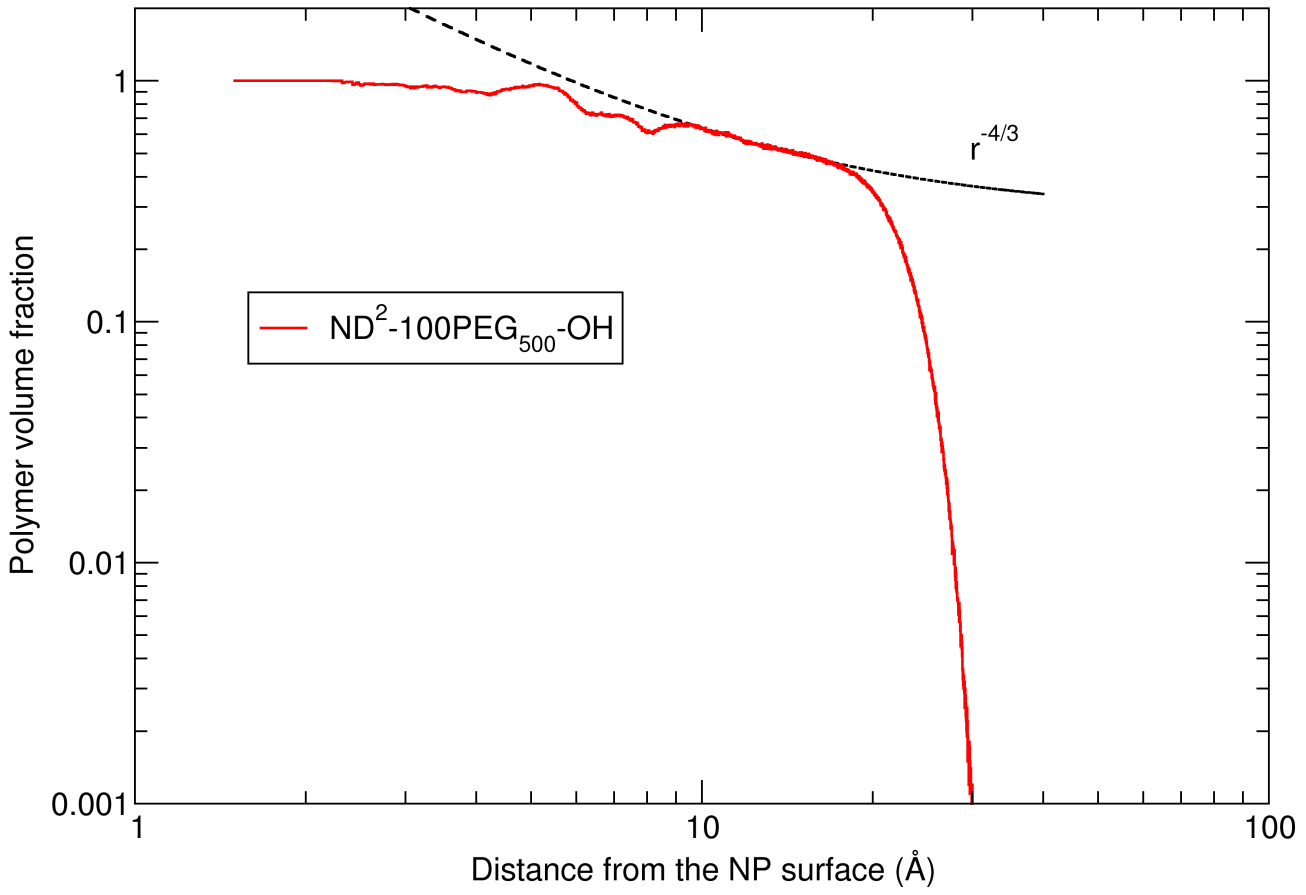

Figure S3. Log-log plot of MD predictions for the polymer volume fraction of the PEG chains from the NP surface towards the bulk-water phase for the ND^{2}-100PEG$_{500}$-OH system. The black dashed line corresponds to the Daoud-Cotton model prediction in the brush regime.

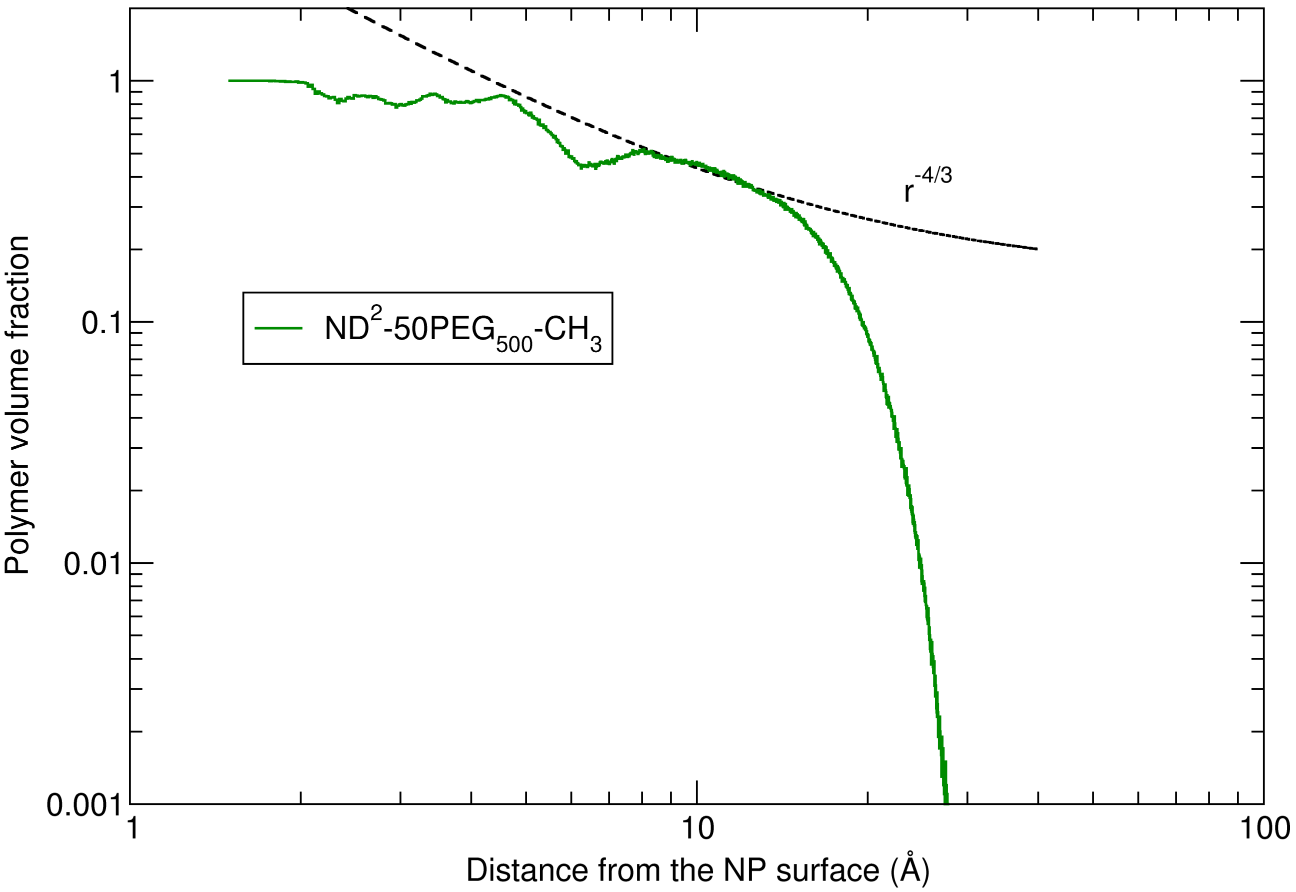

Figure S4. Log-log plot of MD predictions for the polymer volume fraction of the PEG chains from the NP surface towards the bulk-water phase for the ND^2-50PEG_{500}-CH_3 system. The black dashed line corresponds to the Daoud-Cotton model prediction in the brush regime.

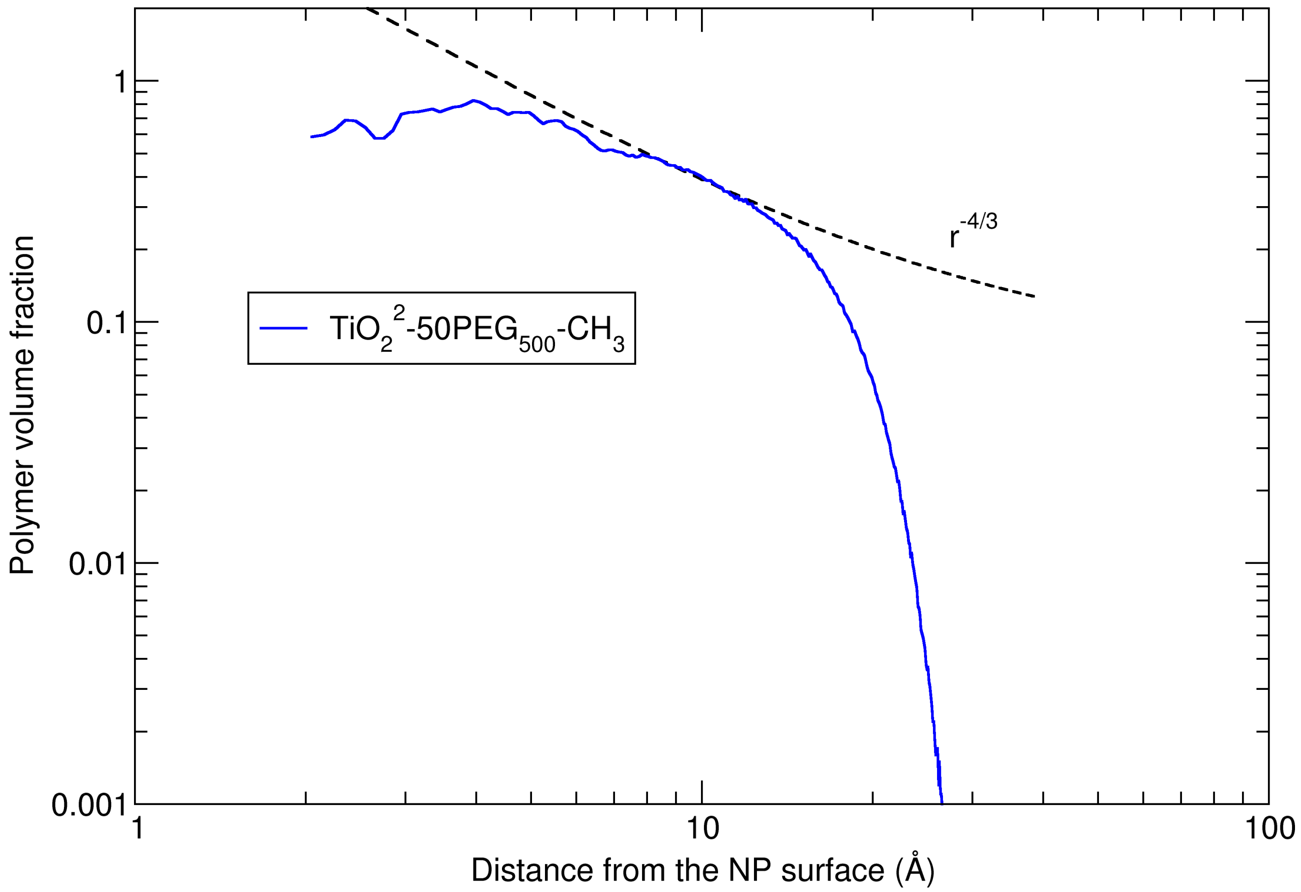

Figure S5. Log-log plot of MD predictions for the polymer volume fraction of the PEG chains from the NP surface towards the bulk-water phase for the TiO_2^2-50PEG_{500}-CH_3 system. The black dashed line corresponds to the Daoud-Cotton model prediction in the brush regime.

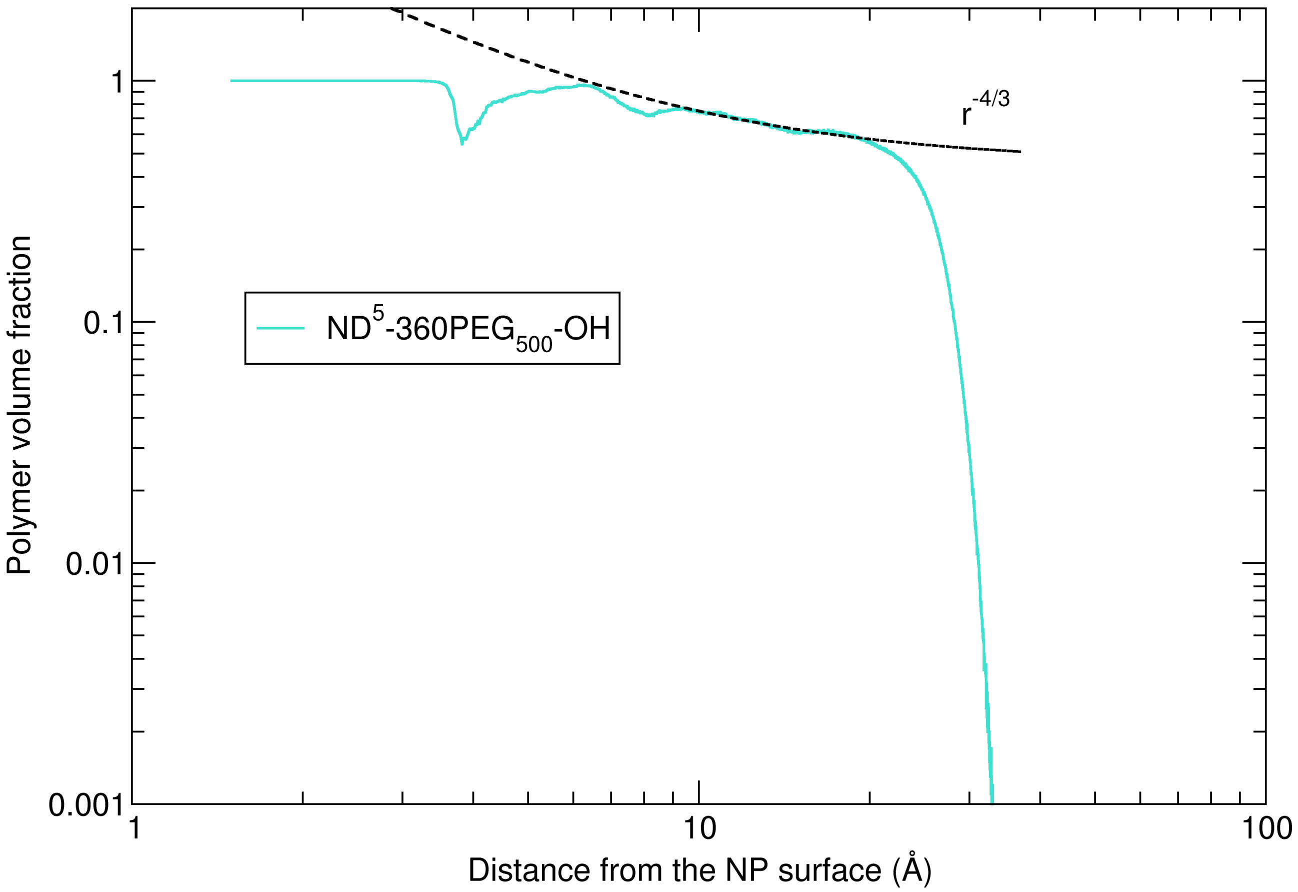

Figure S6. Log-log plot of MD predictions for the polymer volume fraction of the PEG chains from the NP surface towards the bulk-water phase for the ND^5-360PEG_{500}-OH system. The black dashed line corresponds to the Daoud-Cotton model prediction in the brush regime.

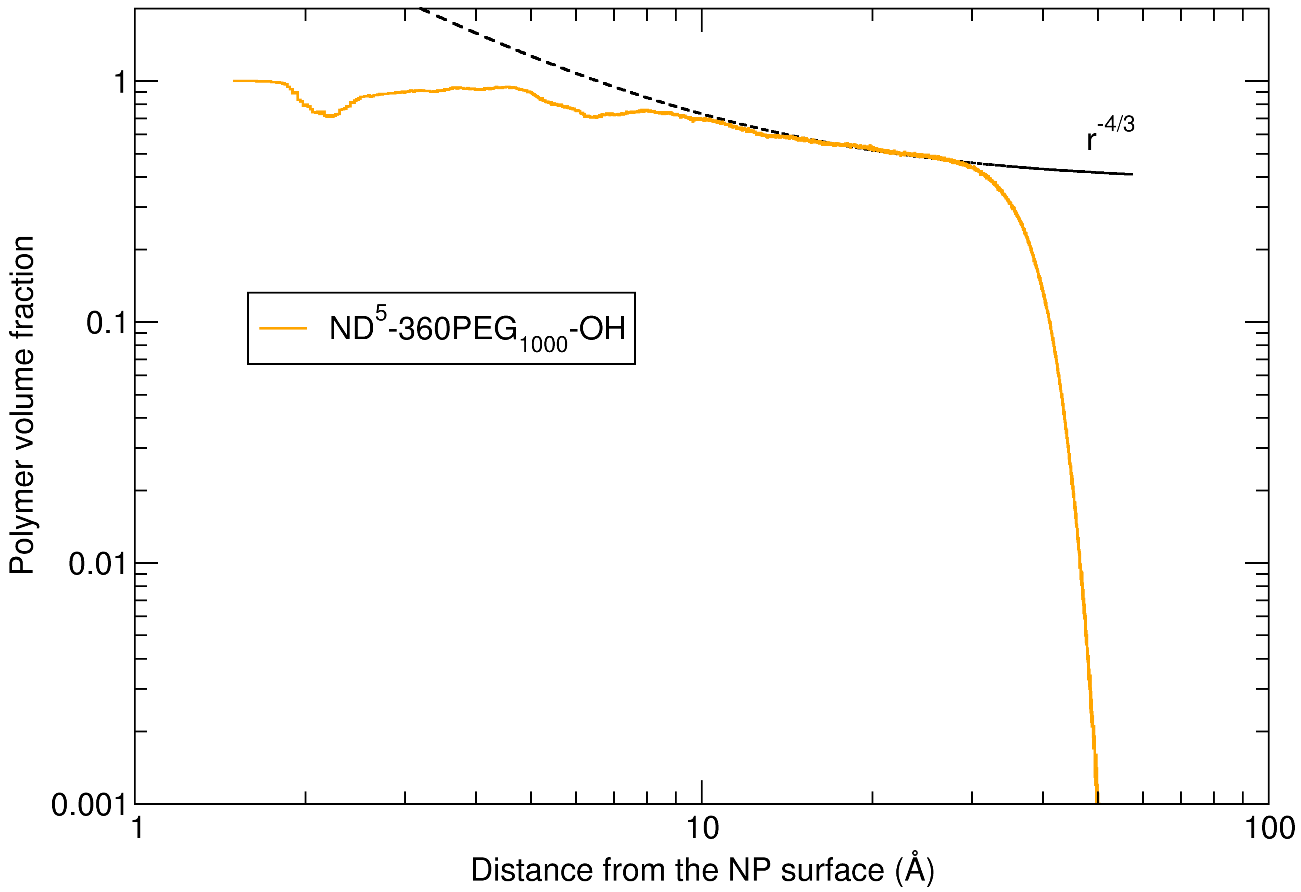

Figure S7. Log-log plot of MD predictions for the polymer volume fraction of the PEG chains from the NP surface towards the bulk-water phase for the ND^5-360PEG$_{1000}$-OH system. The black dashed line corresponds to the Daoud-Cotton model prediction in the brush regime.

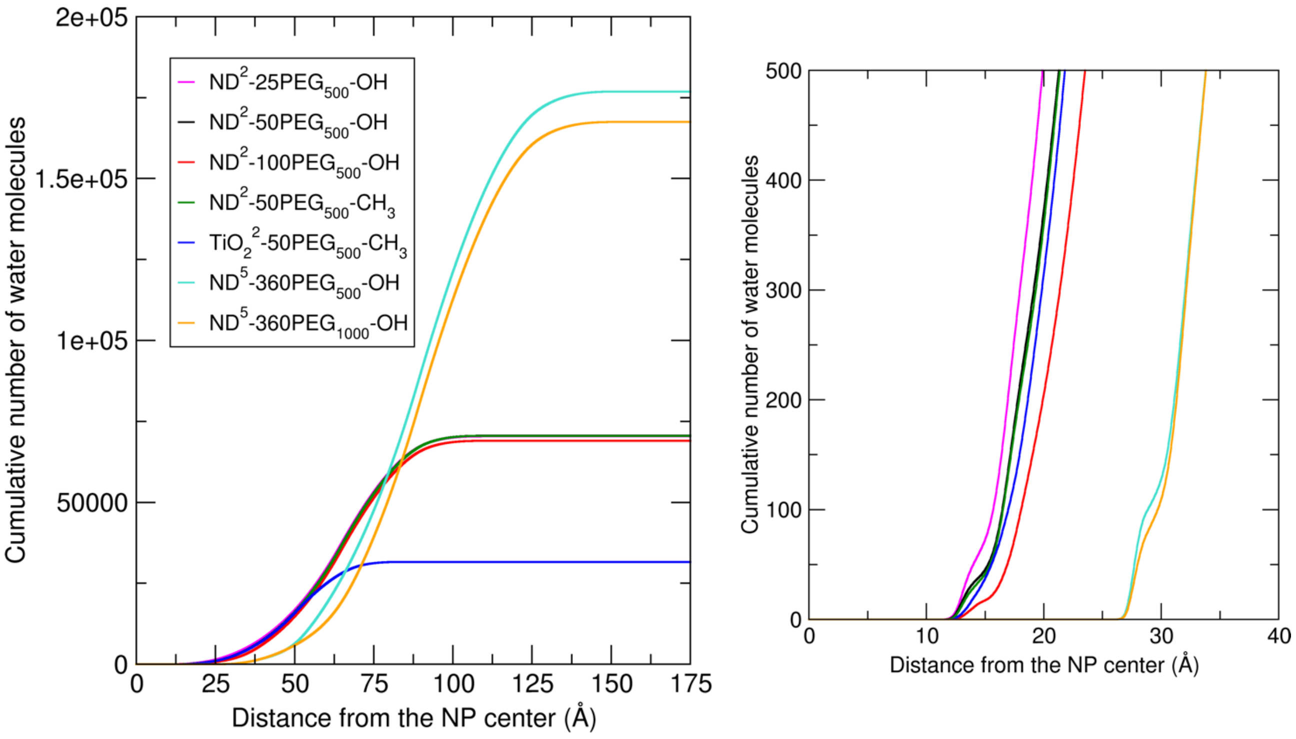

Figure S8. Cumulative number of water molecules as a function of the radial distance from the NP center for all the investigated systems.

Table S3. Average PEG/water H-bonds on the last 10 ns of the 100 ns MD simulations of each system. In parenthesis, the standard deviations are reported.

	ND^2-25PEG_{500}-OH	ND^2-50PEG_{500}-OH	ND^2-100PEG_{500}-OH	ND^2-50PEG_{500}-CH_3	TiO_2^2-50PEG_{500}-CH_3	ND^5-360PEG_{500}-OH	ND^5-360PEG_{1000}-OH
H-bonds number							
PEG/water	78 (±7)	153 (±11)	262 (±15)	151 (±11)	129 (±10)	914 (±28)	1876 (±37)

Table S4. Self-diffusion coefficient (D) estimated on the last 10 ns of the 100 ns-long MD simulations of every investigated system.

System	D (m^2s^{-1})
ND^2-25PEG_{500}-OH	1.3 (±0.5) • 10^{-10}
ND^2-50PEG_{500}-OH	1.2 (±0.3) • 10^{-10}
ND^2-100PEG_{500}-OH	8.3 (±0.3) • 10^{-11}
ND^2-50PEG_{500}-CH_3	1.3 (±0.5) • 10^{-10}
$TiO_2{}^2$-50PEG_{500}-CH_3	2.9 (±0.1) • 10^{-11}
ND^5-360PEG_{500}-OH	2.8 (±0.3) • 10^{-11}
ND^5-360PEG_{1000}-OH	1.4 (±0.2) • 10^{-11}

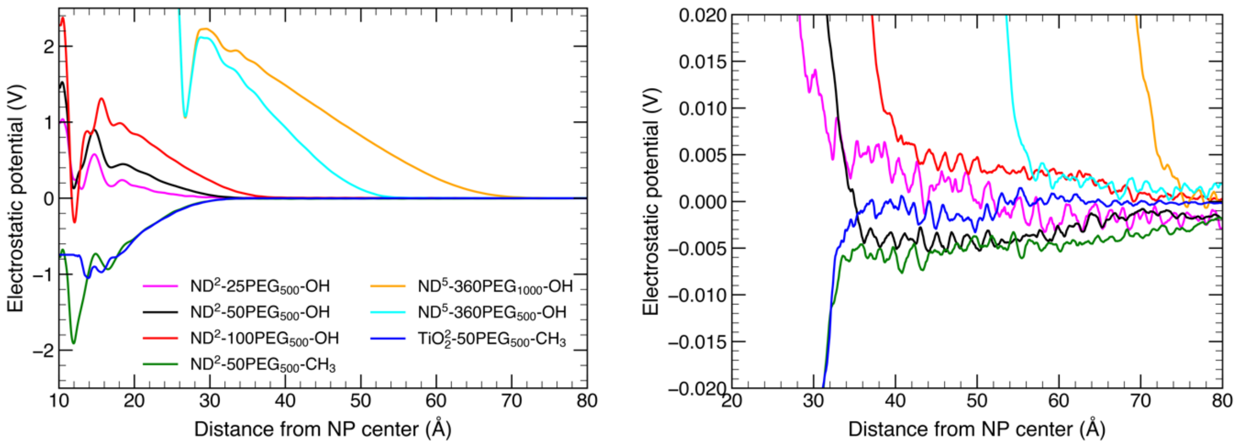

Figure S9. Electrostatic potential as a function of the radial distance from the NP center for all systems.

Table S5. Estimated zeta potential of all systems.

System	Zeta potential (mV)
ND^2-50PEG_{500}-OH	12.1
ND^2-50PEG_{500}-OH	-3.2
ND^2-100PEG_{500}-OH	9.7
ND^2-50PEG_{500}-CH_3	-6.2
TiO_2^2-50PEG_{500}-CH_3	-2.1
ND^5-360PEG_{500}-OH	6.1
ND^5-360PEG_{1000}-OH	3.9

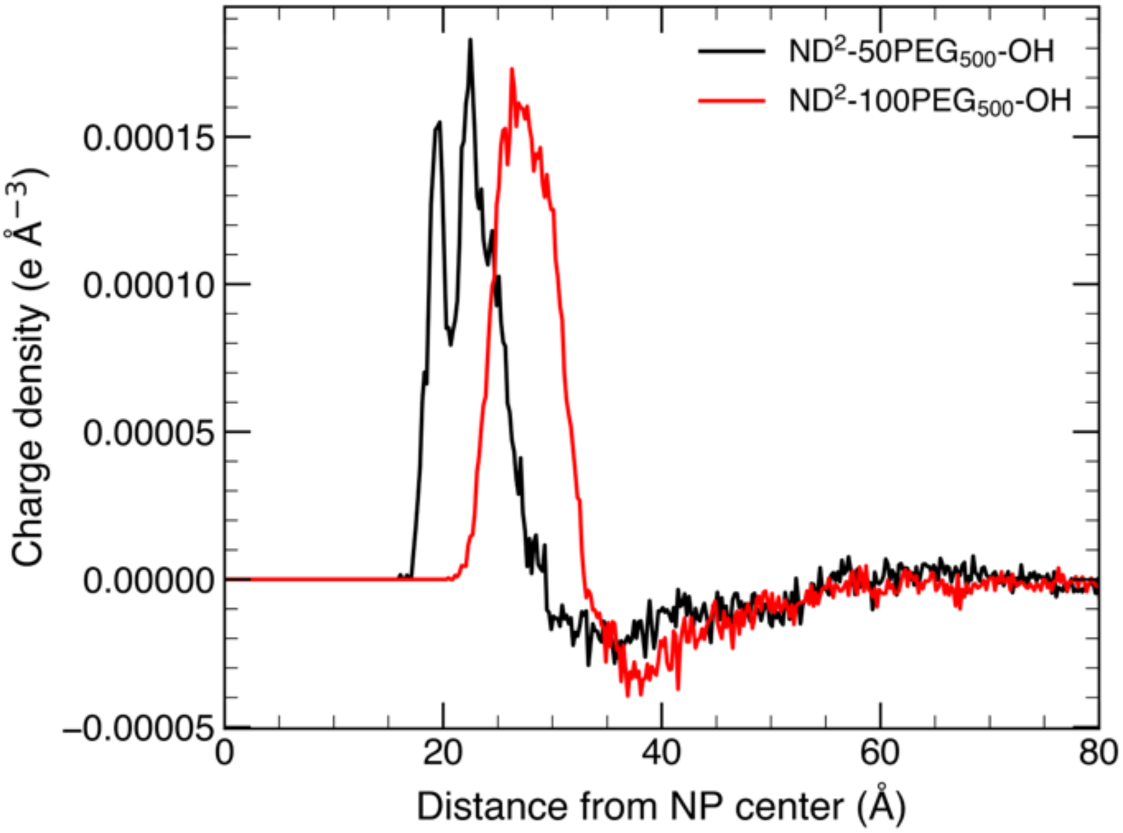

Figure S10. Charge density of the Na^{+} + Cl^{-} ions as a function of the radial distance from the NP center for the ND^{2}-50PEG_{500}-OH (black) and ND^{2}-100PEG_{500}-OH (red) systems.

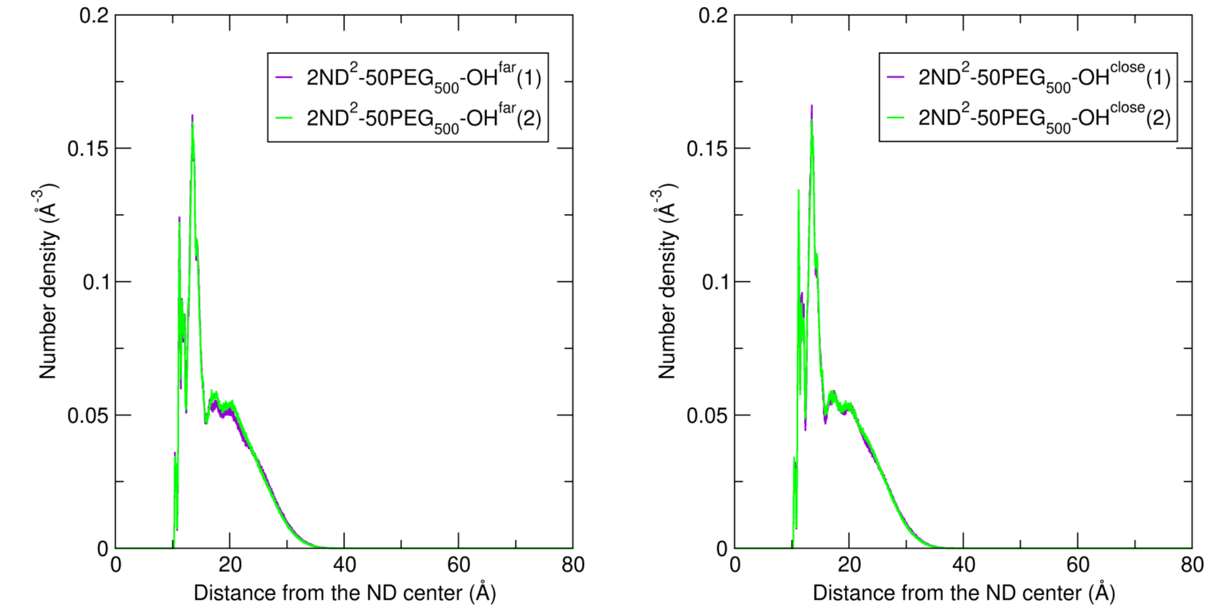

Figure S11. Average number density profiles of the PEG chains computed with respect to the central atom of the ND on the last 10 ns of the 100 ns MD simulations of the $2ND^2$-$50PEG_{500}$-OH^{far} and $2ND^2$-$50PEG_{500}$-OH^{close} systems.

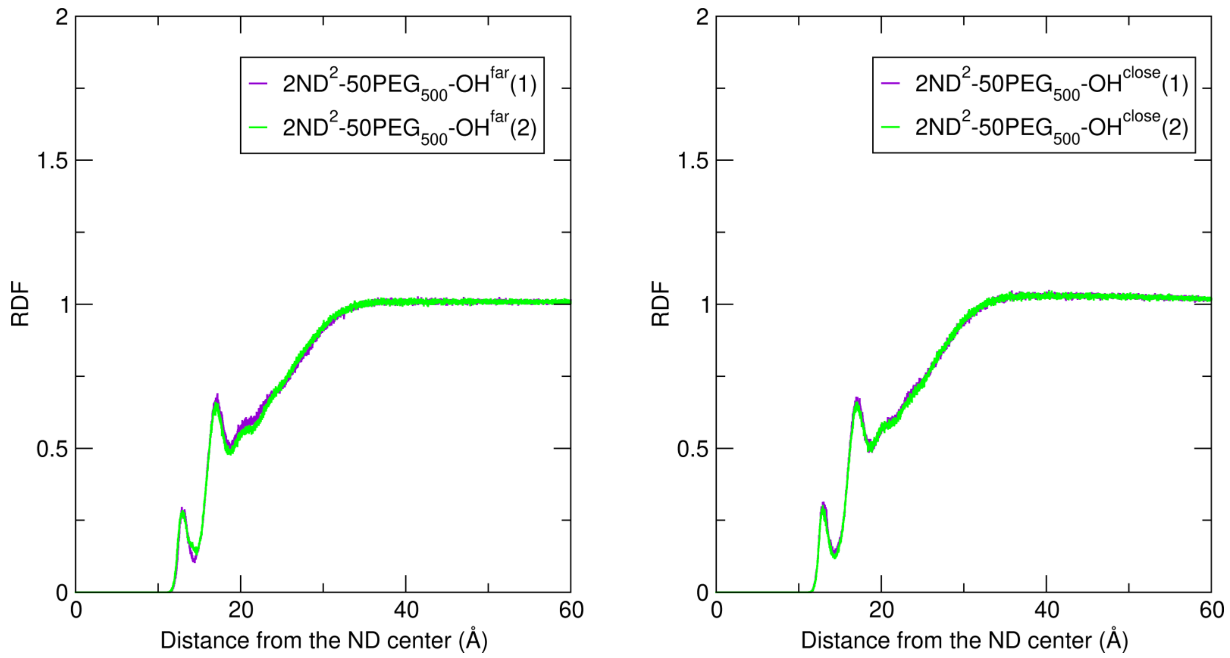

Figure S12. Average RDF of the water molecules computed with respect to the central atom of the ND on the last 10 ns of the 100 ns MD simulations of the $2ND^2$-$50PEG_{500}$-OH^{far} and $2ND^2$-$50PEG_{500}$-OH^{close} systems.

Table S6. Average radius of gyration of the polymer chains, end-to-end distance, PEG-ND distance and thickness of the polymer layer computed on the last 10 ns of the 100 ns MD simulations of the $2ND^2$-$50PEG_{500}$-OH^{far} and $2ND^2$-$50PEG_{500}$-OH^{close} systems. The averages are performed on all the 100 PEG chains of both ND systems. In parenthesis, the standard deviations are reported.

	$2ND^2$-$50PEG_{500}$-OH^{far}	$2ND^2$-$50PEG_{500}$-OH^{close}
R_g (Å)	7.0 (±0.1)	6.9 (±0.1)
$\langle h^2 \rangle^{1/2}$ (Å)	18 (±3)	18 (±2)
$d_{PEG\text{-}ND}$ (Å)	26 (±3)	26 (±2)
thk_{PEG} (Å)	16.40 (±0.03)	16.26 (±0.03)

Table S7. Average non-bonded (electrostatic + vdW) interaction energies and number of hydrogen bonds computed on the last 10 ns of the 100 ns MD simulations of the $2ND^2$-$50PEG_{500}$-OH^{far} and $2ND^2$-$50PEG_{500}$-OH^{close} systems. The averages are performed on all the 100 PEG chains of both ND systems. In parenthesis, the standard deviations are reported.

	$2ND^2$-$50PEG_{500}$-OH^{far}	$2ND^2$-$50PEG_{500}$-OH^{close}
Non-bonded interaction energy (kcal mol^{-1})		
ND/PEG	214 (±11)	212 (±19)
PEG/PEG	-3130 (±162)	-3124 (±38)
ND/wat	-0.04 (±5.24)	-0.55 (±5.26)
PEG/wat	-6032 (±147)	-6030 (±141)
Nanosystem 1-nanosystem 2	-0.0006 (± 0.0012)	-6 (±7)
H-bonds number		
PEG/PEG	2.0 (±1.8)	2.0 (±1.7)
PEG/wat	150 (±11)	151 (±11)

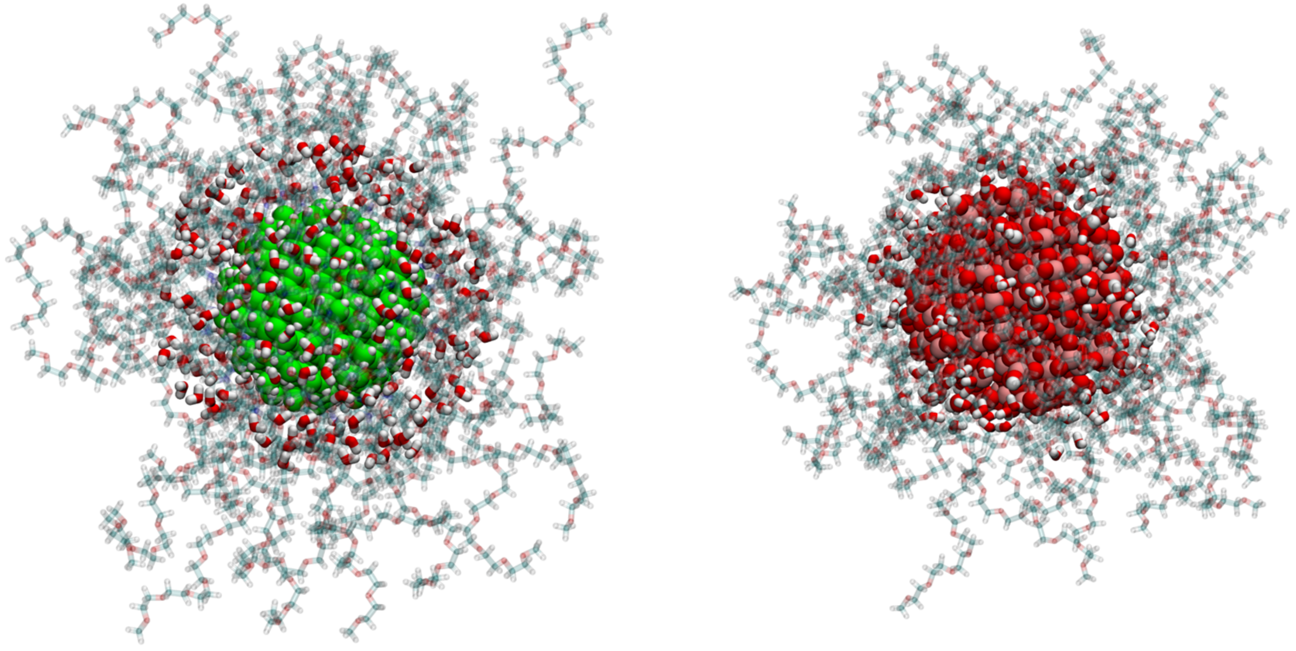

ND^2-50PEG$_{500}$-CH$_3$ **TiO$_2$^{2}-50PEG$_{500}$-CH$_3$**

Figure S13. Water molecules comprising the first and second solvation shells of the ND^2-50PEG$_{500}$-CH$_3$ and the TiO$_2$^{2}-50PEG$_{500}$-CH$_3$ systems. Carbon is shown in cyan, oxygen in red, nitrogen in blue, titanium in pink and hydrogen in white. The carbon atoms of the ND core are shown in green.
